# Advances in single-cell RNA sequencing and its applications in cancer research

**DOI:** 10.1186/s13045-023-01494-6

**Published:** 2023-08-24

**Authors:** Dezhi Huang, Naya Ma, Xinlei Li, Yang Gou, Yishuo Duan, Bangdong Liu, Jing Xia, Xianlan Zhao, Xiaoqi Wang, Qiong Li, Jun Rao, Xi Zhang

**Affiliations:** 1 Medical Center of Hematology, Xinqiao Hospital, State Key Laboratory of Trauma, Burn and Combined Injury, Army Medical University, Chongqing, 400037 China; 2 Jinfeng Laboratory, Chongqing, 401329 China; 3 National Clinical Research Center for Hematologic Diseases, the First Affiliated Hospital of Soochow University, Suzhou, 215006 China

**Keywords:** scRNA-seq, Cancer, Heterogeneity, Tumor microenvironment, Treatment

## Abstract

Cancers are a group of heterogeneous diseases characterized by the acquisition of functional capabilities during the transition from a normal to a neoplastic state. Powerful experimental and computational tools can be applied to elucidate the mechanisms of occurrence, progression, metastasis, and drug resistance; however, challenges remain. Bulk RNA sequencing techniques only reflect the average gene expression in a sample, making it difficult to understand tumor heterogeneity and the tumor microenvironment. The emergence and development of single-cell RNA sequencing (scRNA-seq) technologies have provided opportunities to understand subtle changes in tumor biology by identifying distinct cell subpopulations, dissecting the tumor microenvironment, and characterizing cellular genomic mutations. Recently, scRNA-seq technology has been increasingly used in cancer studies to explore tumor heterogeneity and the tumor microenvironment, which has increased the understanding of tumorigenesis and evolution. This review summarizes the basic processes and development of scRNA-seq technologies and their increasing applications in cancer research and clinical practice.

## Introduction

Cancer is a systemic disease and a major global challenge, that forms and progresses through a series of critical transitions—from premalignant to malignant states: from locally contained to metastatic disease, and from treatment-responsive tumors to treatment-resistant tumors [1–3]. Cancer has been a major challenge because of its clonal heterogeneity and the compositional complexity of the tumor microenvironment (TME) [4]. Tumor heterogeneity and the TME play crucial roles in tumorigenesis, progression, invasion, metastasis, and drug resistance [5–7]. The development of sequencing technologies has allowed the generation of large amounts of molecular data from a single cancer specimen, bringing about the era of precision medicine in clinical oncology [8]. ‘Precision medicine’ requires detailed knowledge of the molecular profile of a patient [9, 10]. Bulk RNA sequencing (RNA-seq) provides limited insights into the clonal composition of tumors and the TME [11]. Several obvious advantages of scRNA-seq over bulk RNA-seq data have been noted, including its ability to characterize the subtypes of cells and the frequency of cell types in each sample, its ability to identify the genes and networks that are activated within each cell or cell type, and the ability to study relationships among cells or cell types [12]. Single-cell RNA sequencing (scRNA-seq) was first reported in 2009 in a study profiling the transcriptome at single-cell resolution, and scRNA-seq is gradually becoming a popular tool used in human cancer research for elucidating disease heterogeneity [4, 13, 14]. ScRNA-seq provides biological information at single-tumor-cell resolution, reveals the determinants of intratumor gene expression heterogeneity, and identifies the molecular bases for the formation of many oncological diseases [15]. Here, we describe the current state of and advances in scRNA-seq technology, summarize its applications in cancer biology research and clinical practice, and propose major avenues for future investigation, with a focus on how this technology can facilitate precision medicine treatment in clinical practice.

## Advances in scRNA-seq

**Fig. 1:**
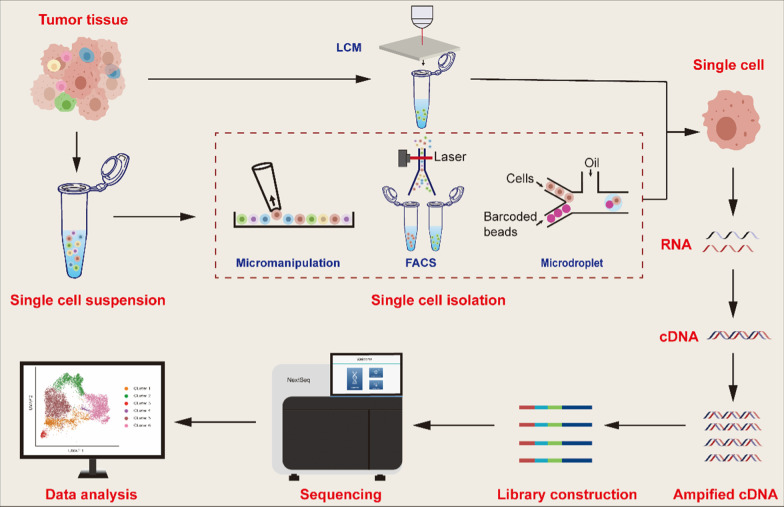
Typical scRNA-seq protocol

A typical scRNA-seq protocol includes several steps: sample acquisition, single-cell isolation, lysis, reverse transcription (RT), complementary DNA (cDNA) amplification, library construction, sequencing, and data analysis [16] (Fig. 1). Although capturing single cells quickly and accurately with high efficiency may seem trivial, it is one of the main challenges of single-cell sequencing [17]. Currently, several methods are utilized to isolate single cells, including manual cell selection [13], limiting dilution [18], laser-capture microdissection (LCM) [19], fluorescence-activated cell sorting (FACS) [20], magnetic activated cell sorting (MACS) [21], and microfluidics [22]. Among these methods, microfluidics has become popular due to its low sample consumption, precise fluid control, and low operating costs [23]. In particular, droplet-based microfluidics (also called microdroplets) is currently the most popular high-throughput platform; in microdroplets, single cells are masked by nanoliter droplets that contain a lysis buffer and barcoded beads using microfluidic and reverse emulsion devices [24].

**Fig. 2:**
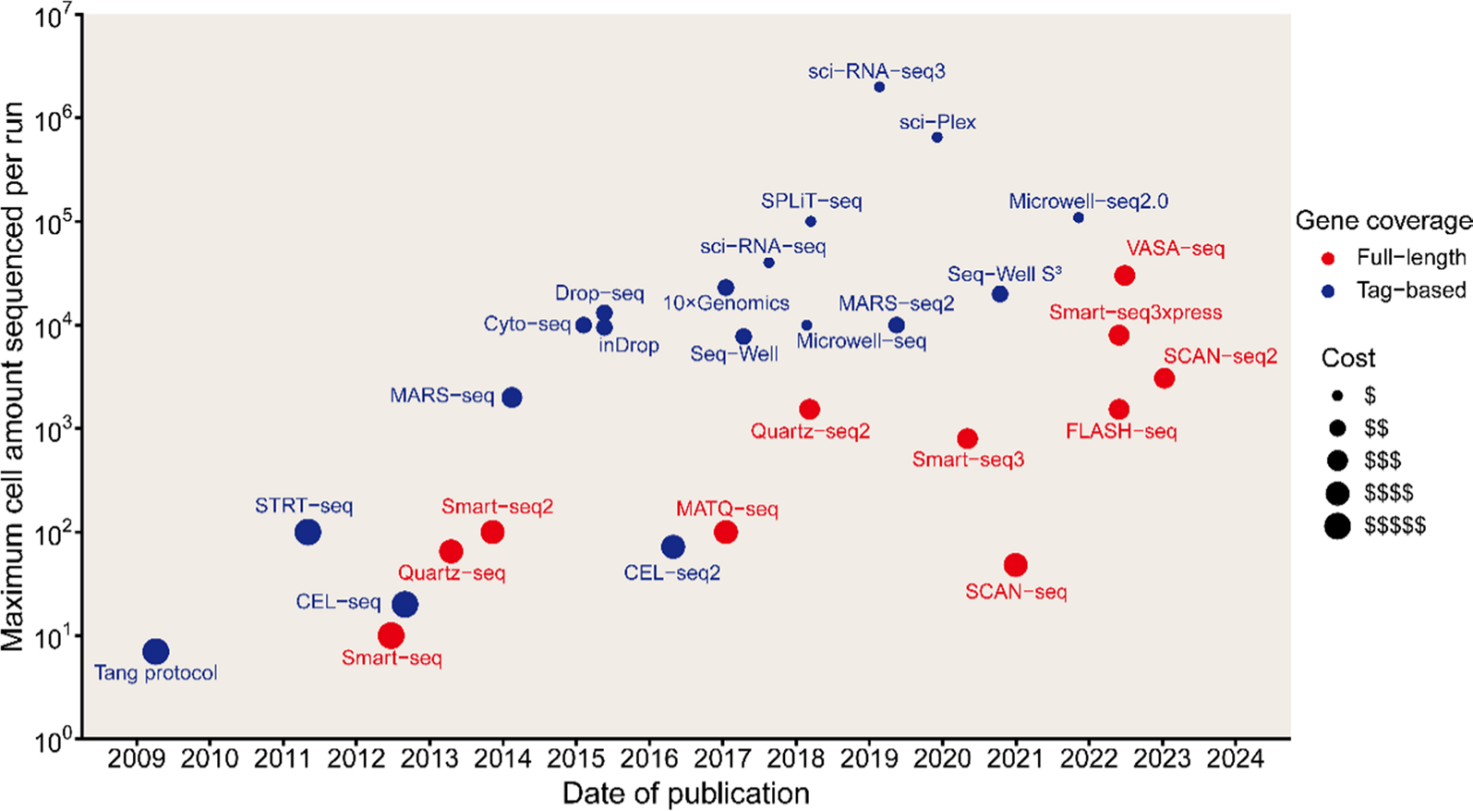
Timeline and throughput of various scRNA-seq methods. Scatterplot depicts the published date and throughput of sequencing for each technology. The color indicates the different gene coverage. Size indicates the cost per sequenced cell of scRNA-seq methods

In general, relevant protocols are classified into two categories: full-length transcript sequencing approaches and 3′/5′-end transcript sequencing approaches (tag-based methods) [25]. Some protocols, such as Quartz-seq [26], Smart-seq [27], Smart-seq2 [28], SUPeR-seq [29], and MATQ-seq [30], can produce full-length transcript sequencing data, while others only capture and sequence the 3′-end, such as CEL-seq [31], CEL-seq2 [32], Drop-seq [33], inDrop [34], 10 × Genomics [35] and Quartz-seq2 [36] or the 5′-end, such as STRT-seq [37]. Compared to 3′-end or 5′-end counting protocols, full-length scRNA-seq methods have incomparable advantages in isoform usage analysis, allelic expression detection, and the identification of RNA editing markers due to their superior transcript coverage [38]. However, full-length scRNA-seq methods are relatively more expensive than contemporaneous tag-based scRNA-seq technology. These methods had their own characteristics and advantages/disadvantages (Table 1, Fig. 2). Next, we will elaborate on the latest advances in scRNA-seq technology by some classic or promising scRNA-seq methods.

**Table 1:** Summary of important scRNA-seq technologies and platforms

Technology	Year	Single-cell isolation	Gene coverage	Library amplification	Throughput	Advantages	Disadvantages	References
10x- Genomics	2017	Droplet	3′ or 5′	PCR	Very high (> 10,000)	High throughput; identifies cells well; ease of use; high cell flux; short library construction cycle; ultra-high capture efficiency	Many steps for DNA library construction; high sample requirements; specialized experimental equipment; non full-length information	[35]
CEL-seq	2012	Micromanipulation	3′	In vitro transcription	Low (1–200)	High specificity and accuracy; first method to use IVT for the amplification	Low efficiency; reduced sensitivity for low expression transcripts	[31]
CEL-seq2	2016	FACS	3′	In vitro transcription	Low (1–200)	High sensitivity; low cost; low hands-on input	Strong 3′ preference; high-abundance transcripts are preferentially amplified	[32]
Cyto-seq	2015	Microwell platform	3′	PCR	High (1000–10000)	Direct analysis of complex samples	Expensive and time-consuming	[233]
Drop-seq	2015	Droplet	3′	PCR	High (1000–10000)	High throughput; low cost; fast amplification; equipment is easily obtained	Low mRNA capture efficiency and low sensitivity	[33]
FLASH-seq	2022	FACS	Full length	PCR	High (1000–10000)	Increased sensitivity and reduced hands-on time compared to Smart-seq3	High manual technical requirements	[62]
inDrop	2015	Droplet	3′	In vitro transcription	High (1000–10000)	High throughput; low cost; strong cell capture capabilities; simplified process	Extremely low cell capture efficiency	[34]
MARS-seq	2014	FACS	3′	In vitro transcription	Median	Reduced amplification bias and labeling errors; high reproducibility	High manual technical requirement	[41]
MARS-seq2	2019	FACS	3′	In vitro transcription	High (1000–10000)	Greatly reduced background noise compared with MARS-seq; minimizes sampling bias and simplifies steps	High manual technical requirement	[44]
MATQ-seq	2017	Micromanipulation	Full length	PCR	Low (100–200)	High sensitivity and accuracy; high transcript capture rate	Inefficient cell lysis	[30, 234]
Microwell-seq	2018	FACS	3′	PCR	High (1000–10000)	High throughput; low cost; high sequencing quality	Presence of 3′ bias; FACS requires skilled operators	[52]
Microwell-seq2	2020	FACS	3′	PCR	High (1000–10000)	Higher utilization of micropores and higher throughput than Microwell-seq; high sensitivity and stability	Presence of 3′ bias; FACS requires skilled operators	[53]
Quartz-seq	2013	FACS	Full length	PCR	Low (1–200)	High sensitivity and high reproducibility	High manual technical requirements	[26]
Quartz-seq2	2018	Droplet	Full length	PCR	High (1000–10000)	High sensitivity; high reproducibility; high accuracy	High manual technical requirements	[36]
SCAN-seq	2020	Dilution	Full length	PCR	Low (1–200)	High sensitivity and accuracy	Low throughput; high cost; high error rate of Nanopore sequencing,	[65]
SCAN-seq2	2023	FACS	Full length	PCR	High (1000–10000)	High throughput and sensitivity; much cheaper than SCAN-seq	Relatively more expensive and lower throughput compared with drop-based scRNA-seq	[66]
sci-Plex	2019	In situ barcoding	3′	PCR	Very high (> 10,000)	Massively multiplex platform; cost-effective; high throughput for drug screening; high resolution	Low UMIs per cell; low cell recovery rate,	[57, 235]
Sci-RNA-seq	2017	In situ barcoding	3′	PCR	Very high (> 10,000)	Minimized perturbation of RNA integrity	Some cell types cannot be defined	[54]
Sci-RNA-seq3	2019	In situ barcoding	3′	PCR	Very high (> 10,000)	Higher throughput; lower cost; nuclei are extracted directly from fresh tissues without enzymatic treatment	Tn5 transposome loaded with specific oligos is not commercially available; reduced gene detection rate compared with 10 × Genomics	[56, 236]
Seq-Well	2017	Microwell platform	3′	PCR	High (1000–10000)	Easy-to-use; portable; low-cost; efficient cell lysis and transcriptome capture	Low cell capture efficiency	[48]
Seq-Well S^3^	2020	Microwell platform	3′	PCR	Very high (> 10,000)	High-throughput; high-fidelity	Short cDNA; presence of 3 'bias	[50]
SMART-seq	2012	FACS	Full length	PCR	Low (1–200)	Full-length coverage	Low efficiency; limited throughput and read coverage	[27]
SMART-seq2	2013	FACS	Full length	PCR	Median (100–1000)	Higher sensitivity and higher transcription coverage; cell capture visualization; low amplification bias; low variability and low noise; analysis of rare cell populations	No early multiplexing; low reproducibility; extremely high manual technical requirements	[28]
SMART-seq3	2020	FACS	Full length	PCR	Median (100–1000)	Much more sensitive and higher throughput than SMART-seq2; provides cost-effective RNA analysis at isoform resolution	No early multiplexing; extremely high manual technical requirements	[59]
Smart-seq3xpress	2022	FACS	Full length	PCR	High (1000–10000)	Shortens and streamlines the Smart-seq3 protocol to substantially reduce reagent use and increase cellular throughput	No early multiplexing; extremely high manual technical requirements	[60]
SPLit-seq	2018	In situ barcoding	3′	PCR	Very high (> 10,000)	Low cost and minimal equipment requirements; no need for cell isolation; suitable for fixed cells and fixed nuclei	Not enough genes	[55]
STRT-seq	2011	FACS	5′	PCR	Median (100–1000)	Multiplexable; can be used to study many different single cells at a time; reduced cross-contamination	PCR biases; nonlinear PCR amplification	[37]
VASA-seq	2022	Plate-based formats and droplet microfluidics	Full length	PCR	High (1000–10000)	The only single-cell sequencing technology that combines high sensitivity, full-length transcriptome coverage; with high throughput; cost-effective; compatible with all sample types	Integration with other datasets (which necessitates batch corrections), and creation of specialized data analysis pipelines	[67, 68]

### Tag-based methods

The main advantage of tag-based methods is that these can be combined with unique molecular identifiers (UMIs), which can reduce overall costs and labor, enable the multiplexing of more samples, and improve gene-level quantification and throughput. However, tag-based methods have relatively low sensitivity as mappable reads are restricted to one end of the transcript. Thus, tag-based methods are mostly used for gene expression quantification and cannot be utilized for isoform identification or splicing [39, 40].

#### CEL-seq and CEL-seq2

Linear amplification by in vitro transcription (IVT) is preferable to exponential amplification by PCR. CEL-seq (cell expression by linear amplification and sequencing) is the first scRNA-seq protocol that uses IVT for linear amplification of RNA from single cells [31], which is a sensitive, accurate, and reproducible single-cell transcriptomics method. However, the experiment procedure is complicated and time-consuming. The throughput is low and it has a 3′ bias. Four years later, the same team created CEL-seq2 by combining with Fluidigm’s C1 system, which is the first single -cell, on-chip barcoding method with more time- and cost-efficient compared with CEL-seq [32]. Moreover, CEL-seq2 increases accuracy with the addition of 5-base UMI for labeling and significantly improves RT efficiency, resulting in increased assay sensitivity.

#### MARS-seq and MARS-seq2

MARS-seq (massively parallel single-cell RNA-sequencing) is an automated high-throughput method of CEL-seq and was developed to explore cellular heterogeneity within the immune system by assembling an automated experimental platform that enables RNA profiling of cells sorted from tissues using flow cytometry [41]. MARS-seq utilizes IVT as the amplification method instead of PCR to quantify the mRNA levels with less amplification noise and reduce hands-on time with the ability to pool many samples before amplification. At the same time, the requirement for barcoding limits coverage to only the 3′ or 5′ ends of the transcripts [42, 43]. Shaul and colleagues developed a novel MARS-seq2.0 based on the MARS-seq protocol, whose experimental improvements refer to lowering of RT volume, optimization of lysis buffer, reduction of RT primer concentration, optimization of RT primer composition, primer removal by exonuclease I, optimization of second-strand-synthesis enzymes, and optimization of barcoded ligation adaptor [44]. Thus, MARS-seq2.0 has a comprehensive improvement including throughput, robustness, noise reduction, and cost reduction. Optimization of the conditions indicated above resulted in a sixfold reduction in the cost of library production (from $0.65 to $0.10 per cell) and reduced the background level (from 10–15% to 2%). MARS-seq2.0 allows efficient sequencing of 8,000–10,000 cells in a single run and has a negligible amount of doublet cells (< 0.2%; 2 out of 1,041 cells) and provides high confidence in cell identity. MARS-seq2.0 only takes 2–3 days from cell sorting to a ready-to-sequence library. Sequencing and processing the data through the analytical pipeline take another 1–2 days. The drawback of this method is the premature termination of reverse transcription which significantly reduces transcript coverage at the 5′ end [23].

#### *Drop-seq, in Drop-seq and 10* × *Genomics*

Currently, the most popular high-throughput platform is based on droplet-based microfluidics (microdroplets) [45]. In 2015, two blockbuster droplet-based scRNA-seq methods, known as Drop-seq and inDrop, were identified [33, 34]. Drop-seq and inDrop share similar strategies in generating droplets, isolating single cells through on-bead primers with barcodes, and correcting bias by applying UMIs [46]. In 2017, the commercial sequencing platform 10 × Genomics was successfully developed based on the above techniques, enabling a significant increase in cell throughput and a considerable reduction in single-cell sequencing costs [14, 35]. Zhang et al. compared the three most widely used droplet-based high-throughput scRNA-seq systems using the same cell sample and a unified data processing pipeline to reduce bias in experimental design and data analyses [47]. The instrument of 10 × Genomics costs more than $50,000 and the per-cell cost is about $0.50, even without considering the sequencing cost or instrument depreciation. Building up the whole system of Drop-seq costs less than $30,000 and the experimental cost is about $0.10 per cell. The instrument cost of inDrop is comparable to that of 10 × Genomics, and the per-cell cost is about half that of 10 × Genomics. Generally, all three systems offer satisfactory transcript detection efficiency, and higher efficiency is associated with higher experimental costs. When the sample is abundant, Drop-seq can be more cost-efficient. In contrast, when the detection of low-abundance transcripts is optional, or a custom protocol is desired, inDrop becomes a better choice. As a more mature commercialized system, 10 × Genomics generally requires less time, has higher molecular sensitivity and precision, and is accompanied by less technical noise. By rule of thumb, 10 × Genomics is currently a safe choice for most applications and has been used in cancer research most widely. However, these techniques can only identify the 3′ or 5′ end sequence of transcripts and have a limited depth of sequencing.

#### ***S***eq-Well and*** Seq-Well S***^***3***^

Seq-Well resembles its predecessor Drop-seq but surpasses it. Using similar chemistry but without droplets, cells are efficiently loaded by gravity into picowells where single cells and uniquely barcoded poly(dT) mRNA beads are co-confined with a semipermeable membrane, which reduces the need for peripheral equipment, decreases dead volumes and facilitates parallelization [48, 49]. Seq-Well overcomes key cost, portability, and scalability limitations associated with reverse-emulsion droplets-based cell capture and barcoding methods like Drop-seq by combining the throughput and cost-effectiveness of Drop-seq with the simplicity and sampling efficiency of picowells [49]. High-throughput scRNA-seq methodologies recover less information per cell than low-throughput strategies. To achieve the goal of both high fidelity and high throughput, this research team created Seq-Well S^3^ (‘‘Second-Strand Synthesis’’), which incorporates a second-strand-synthesis step after reverse transcription to add a second PCR priming site [50]. This modification allows for the recovery of cDNA that is reverse transcribed but for which the template switch reaction failed. Seq-Well S^3^ increases the efficiency of transcript capture and gene detection compared with Seq-Well by up to 10- and fivefold, respectively. However, a limitation of Seq-Well S^3^ is that the size of the cDNAs after second-strand synthesis was shorter than that obtained in Seq-Well or Drop-seq, which decreases the utility of Seq-Well S^3^ for certain downstream applications that seek information from full-length transcripts or their 5’ ends.

#### Microwell-seq and Microwell-seq2.0

Microwell-seq uses agarose microarray to trap individual cells and fabrication of the agarose microarray is a high-throughput, convenient, and low-cost scRNA-seq platform with advantages of low batch effects and high cell-type compatibility [51, 52]. It can capture 5–10 thousand individual cells by agarose plates with 10^5^ microwells in a single experiment [45]. Microwell-seq produces high-fidelity single-cell libraries with no more than 1.2% cell doublets. Approximately 6,500 genes and 55,000 transcripts can be detected by saturated sequencing [52]. Microwell-Seq contributes to cellular hierarchy construction and clonal heterogeneity deciphering in normal bone marrow and acute myeloid leukemia [7]. Combining in-cell RT and Microwell-seq, Chen et al. established Microwell-seq2.0 for cost-effective and high-throughput screening (HTS) with single-cell transcriptional profiling [53]. Compared with Microwell-seq, Microwell-seq2.0 has a higher sensitivity, speeds up the process, and drastically reduces the cost. An agarose plate of Microwell-seq2.0 with 70,000 wells can contain up to 700,000 individual cells in a single experiment, which tremendously improves the throughput. This method may pave the way for a more cost-effective multi-dimensional and high-throughput drug screening assay.

#### Series of sci-RNA-seq, SPLiT-seq and sci-Plex

Instead of isolating single cells within physical compartments, single-cell combinational indexing RNA sequencing (sci-RNA-seq) in 2017 was successively developed using a two-step combinatorial indexing strategy, a method using split-pool barcoding of nucleic acids to uniquely label a large number of single molecules or single cells [54]. The sci-RNA-seq can generate ~ 4 × 10^4^ single-cell transcriptomes in one experiment through a library construction completed by a single person in 2 days, for $0.03 to $0.20 per cell. Later, a similar method, split-pool ligation-based transcriptome sequencing (SPLiT-seq), was developed, which requires four rounds of split-pool barcoding [55]. Like sci-RNA-seq, it does not require additional pretreatment and uses its cells as a compartment for subsequent sequencing operations. This method enables transcriptional profiling of hundreds of thousands of fixed cells or nuclei in a single experiment using only basic laboratory equipment with a library preparation cost of ~ $0.01 per cell. In addition, the quality of scRNA-seq data obtained was similar to that obtained with Drop-seq and inDrop [14]. In 2019, Cao et al. [56] proposed sci-RNA-seq3 by optimizing their previously established sci-RNA-seq through four aspects of nuclei extraction, the third level of indexing, individual enzymatic reactions, and cell sorting. This method profiled the transcriptomes of around 2 million cells derived from 61 embryos staged between 9.5 and 13.5 days of gestation in a single experiment. The library preparation can be completed through the intensive effort of a single researcher in one week at a cost of less than $0.01 per cell. In 2020, Srivatsan et al. [57] introduced a new sample labeling (hashing) strategy that relied on labeling nuclei with unmodified single-stranded DNA oligos. They combined nuclear hashing and sci-RNA-seq into a single workflow for multiplex transcriptomics in a process called “sci-Plex.” They applied sci-Plex to screen three cancer cell lines exposed to 188 compounds and profiled ~ 650,000 single-cell transcriptomes across ~ 5000 independent samples in one experiment. The ease and low cost of oligo hashing, coupled with the flexibility and exponential scalability of single-cell combinatorial indexing, would facilitate the goal of a comprehensive, high-resolution atlas of cellular responses to pharmacologic perturbations. In summary, these methods have high cell labeling efficiencies and can drastically reduce the cost of library preparation. However, the operations of these methods are tedious and cell fixation will result in the loss of transcripts and impaired sensitivity.

### Full-length methods

Compared to methods only capturing and sequencing the 3′ or 5′ ends of the cDNAs, protocols capable of full-length transcription are more suitable for alternative splicing pattern analyses, allelic expression detection, and RNA editing identification owing to their superiority of transcript coverage [58]. The full-length scRNA-seq methods represented by Smart-seq2 are also widely used in tumor research.

#### Series of Smart-seq and FLASH-seq

The series of Smart-seq are full-length and plate-based scRNA-seq methods and evolve continually. Smart-seq published by Ramsköld et al. in 2012 became the first to apply to tumor cells to identify distinct gene expression patterns [27]. This method has been further refined to develop Smart-seq2, Smart-seq3, and Smart-seq3xpress techniques by the research group. Smart-seq2 improves throughput, sensitivity, accuracy, and full-length coverage, and reduces cost by refining reverse transcription, template switching, and preamplification [28]. With these improvements, Smart-seq2 is suitable for discovering variable splicing events and allele-specific expression. Smart-seq2 has been seen as the gold standard method of scRNA-seq and has been used in various cancer research. Smart-seq3 combines full-length transcriptome coverage with a 5’ UMI RNA counting strategy that enables in silico reconstruction of thousands of RNA molecules per cell [59]. Smart-seq3 greatly increases sensitivity compared to Smart-seq2, typically detecting thousands more transcripts per cell. Moreover, this method costs about €0.5–€1 per sequencing-ready cell library in 384-well plates with moderate cellular throughput. In this way, Smart-seq3 can count RNAs at allele and isoform resolution for large-scale applications across cells. High cellular throughputs usually sacrifice full-transcript coverage and sensitivity. Smart-seq3xpress which miniaturizes and streamlines the Smart-seq3 protocol reduces the material and resources needed to construct Smart-seq3xpress single-cell libraries by ten-fold and increases cellular throughput [60]. The sequencing-ready libraries can be generated in a single workday. Therefore, high-sensitivity Smart-seq3xpress with isoform-specific and allele-specific resolution can, for the first time, be performed at a scale suitable for large-scale cell atlas building. Building upon the existing Smart-seq2/3 workflows, Hahaut et al. developed FLASH-seq (FS), a new full-length scRNA-seq method capable of detecting a significantly higher number of genes than previous versions, requiring limited hands-on time (~ 4.5 h) and with a great potential for customization [61, 62]. Based on FS, this group constructed another two protocols: FLASH-seq low-amplification (FS-LA) and FLASH-seq with UMIs (FS-UMI). FS-LA protocol is cheaper than FS and requires < 1 h of hands-on time without sacrificing performance. FS-UMI builds upon the same principle as Smart-seq3 and introduces UMIs for molecule counting and isoform reconstruction. The newly designed template-switching oligonucleotide (TSO) contains a 5-bp spacer, which allows the generation of high-quality data while minimizing the number of strand-invasion artifacts. The cost of per cell is lower than other commercial and noncommercial methods and comparable to Smart-seq3 (< $1). FS has the potential to become the tool of choice when looking for an efficient, robust, modular, affordable, and automation-friendly full-length scRNA-seq protocol. However, a common limitation shared among Smart-seq2/3 and FLASH-seq is that all use an oligo dT-based strategy for priming exclusively polyadenylated RNAs, thus neglecting other potentially relevant RNA species such as microRNAs (miRNAs), piwi-interacting RNAs (piRNAs), and non-polyadenylated long non-coding RNAs (lncRNAs) [61].

#### Quartz-seq and Quartz-seq2

To comprehensively and quantitatively detect gene expression heterogeneity, another full-length scRNA-seq approach termed Quartz-Seq was developed immediately after Smart-seq in 2013 [26]. Quartz-Seq is a simple, sensitive, reproducible, and highly quantitative scRNA-seq approach. By optimizing the five steps of single-cell collection, cell barcoding, the pooling of cell-barcoded cDNA, whole-transcript amplification, and library preparation, a higher throughput Quartz-Seq2 was developed [36]. It can analyze cells numbering up to 1536 that are pooled together in a single sample and effectively uses limited sequence reads. In a study, the researchers investigated the ability of the 13 scRNA-seq methods to draw cell maps from six aspects: genetic detection, marker expression, clusterability, mappability, clusterability (integrated), and mixability. The findings revealed that the Quartz Seq2 method outperformed other schemes, including 10 × Genomics and Smart-seq2, exhibiting the highest benchmarking score and thus demonstrating superior accuracy [63].

#### SCAN-seq and SCAN-seq2

There are still many questions that cannot be addressed by them due to the short read lengths of next-generation sequencing (NGS) platform-based scRNA-seq. Further development of long-read RNA sequencing, known as third-generation sequencing, can be used to generate full-length cDNA transcripts with a minimum number of false-positive splice sites and capture great diversity of transcript isoforms [64]. Fan et al. [65] developed a novel scRNA-seq technology based on third-generation sequencing (TGS) platform (single-cell amplification and sequencing of full-length RNAs by Nanopore platform, SCAN-seq). SCAN-seq exhibits high sensitivity and accuracy comparable to NGS platform-based scRNA-seq methods. Recently, the research group refine SCAN-seq to develop SCAN-seq2, especially on throughput and cost [66]. SCAN-seq2 can sequence up to 3072 single cells for one sequencing run and the cost of an individual cell is reduced to about $3 for a sequencing run of 960 cells, which is 20 times cheaper than SCAN-seq (about $60 for each cell). Compared with other published scRNA-seq methods based on the TGS platform, SCAN-seq2 also exhibits high throughput and high sensitivity simultaneously. SCAN-seq2 proves to be a new promising tool for single-cell full-length transcriptome research, which can be used to study different biological systems at single-cell and individual RNA isoform resolution and help understand the complex mechanisms of many diseases.

#### VASA-seq

The majority of techniques used for single-cell transcriptome sequencing focus on amplifying the termini of polyadenylated transcripts, resulting in a limited representation of the entire cellular transcriptome. This limitation poses challenges in detecting various types of transcripts, such as long non-coding, short non-coding, and non-polyadenylated protein-coding transcripts, and hinders alternative splicing analysis. To address this issue, Salmen et al. [67] developed a full-length VASA-seq method that allows for the detection of complete transcriptomic atlases in single cells, including alternative splicing and non-coding transcripts in single cells, which is enabled by fragmenting and tailing all RNA molecules subsequent to cell lysis. The method is compatible with both plate-based formats and droplet microfluidics. Additionally, the reduction in reagent expenses resulting from the downsizing of droplets and the elimination of dependence on commercially available kits in the VASA-drop methodology will facilitate cost-effective, extensive transcriptomic profiling on a large scale. This profiling can be achieved at an approximate cost of $0.11 per cell for libraries ready for sequencing. The VASA-plate method incurs a library preparation cost of $0.98. However, the routine implementation of VASA-seq is impeded by various practical challenges. These challenges include the need to expand the coverage of RNA molecules, such as miRNAs, achieving a balance between the length of poly(A) tails and RNA fragments, optimizing steps for ribosomal RNA depletion, estimating splicing node inclusion rates in situations of low coverage per cell, integrating with other datasets requiring batch corrections, and developing specialized data analysis pipelines [68]. In conclusion, it can be stated that VASA-seq remains the sole technology that effectively integrates exceptional sensitivity, comprehensive coverage of total RNA, and efficient high throughput. Furthermore, it is anticipated that VASA-seq will offer further analytical perspectives by incorporating gene regulation and splicing pattern localizations across various tissues. [67, 68].

### Spatially resolved transcriptomics

Recently, methods for spatially resolved transcriptomics (SRT) are developed by integrating scRNA-seq with cellular locations for generating tissue-wide landscapes of single-cell transcriptomes and identifying cellular composition and molecular architecture within the tissues, which could overcome the limitation of loss of spatial and morphologic information among the cataloged populations of cells [24, 48, 49]. It has also been applied to dissect the spatial heterogeneity of human liver cancer [50], breast cancer [51–53], glioblastoma [54], colorectal cancer (CRC) [55], and ovarian cancer [56].

## Applications of scRNA-seq in human cancer biology

**Fig. 3:**
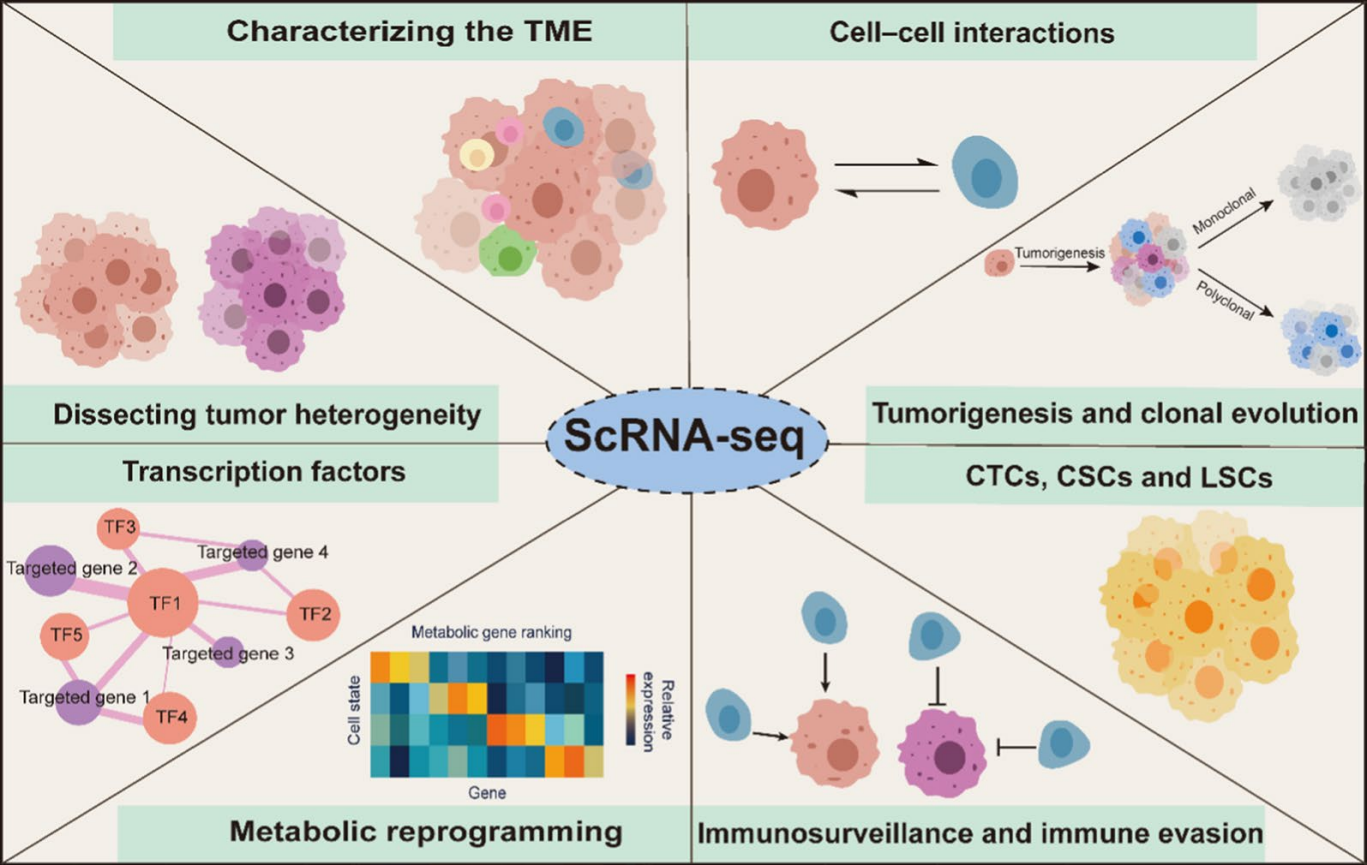
Applications of scRNA-seq in human cancer biology

In human cancer research, scRNA-seq has been widely used to study heterogeneity, the TME, gene expression profiles, transcriptome profiles, and cell‒cell interactions, and other biology related to cancer research (Fig. 3). The applications of scRNA-seq in cancer research are explored and discussed in this section.

### Dissecting tumor heterogeneity

Tumor heterogeneity, including intertumor (tumor by tumor) and intratumor (within a tumor) heterogeneity (ITH), is a key characteristic of malignant tumors and a significant obstacle in cancer treatment and research [69, 70]. Recognizing tumor heterogeneity is key for further understanding and treating cancers. Almost all single-cell studies of cancer have focused on or studied tumor heterogeneity. Dissecting tumor heterogeneity with scRNA-seq has been used to facilitate cancer diagnosis [71] and prognosis prediction [72], increase the understanding of disease progression and cancer metastasis [73, 74], and guide therapy [75] (Table 2). Recently, to deepen the understanding of tumor heterogeneity, single-cell sequencing technologies are often combined with other technologies, such as single-cell genomics, single-cell proteomics, single-cell epigenomics, etc. Other spatial omics, such as spatial transcriptomics, spatial proteomics, and spatial metabolomics have or will be combined with the scRNA-seq to deepen and broaden our understanding of tumor heterogeneity.

**Table 2:** Key findings related to tumor heterogeneity among various tumors using scRNA-seq

Tumor	Year	Species	Protocol	Accession number (custom database if available)	Key findings	References
Lung cancer	2020	Human	10 × Genomics	EGAD00001005054	Identified a cancer cell subtype deviating from the normal differentiation trajectory and dominating the metastatic stage, and revealed potential diagnostic and therapeutic targets in cancer-microenvironment interactions	[156]
	2020	Human	Smart-seq2	NCBI BioProject #PRJNA591860	Identified that individual tumors and cancer cells exhibit substantial molecular diversity and that tumor microenvironment cells exhibit marked therapy-induced plasticity	[157]
	2022	Human	STRT-seq	HRA000270	Provided novel insights into the tumor heterogeneity of NSCLC in terms of the identification of prevalent mixed-lineage subpopulations of cancer cells with combined SCC, ADC, and NET signatures and offered clues for potential treatment strategies in these patients	[158]
Gastric cancer	2020	Human	10 × Genomics	PRJEB40416	Highlighted response heterogeneity within MSI-H gastric cancer treated with pembrolizumab monotherapy; supported the potential of extended baseline and early on-treatment biomarker analyses to identify responders	[95]
	2021	Human	10 × Genomics	EGAS00001004443	The links between tumor cell lineage/state and ITH were illustrated at the transcriptome, genotype, molecular, and phenotype levels	[72]
	2021	Human	10 × Genomics	HRA000051	A panel of differentiation-related genes revealed large differences in differentiation degree within and between tumors	[80]
Liver cancer	2022	Human	Seq-Well S^3^	GSE186975	Identified five hepatoblastoma tumor signatures that may account for the tumor heterogeneity observed in this disease, and used patient-derived hepatoblastoma spheroid cultures to predict differential responses to treatment based on the transcriptomic signature of each tumor	[237]
Esophageal cancer	2022	Human	10 × Genomics	GSE196756	Revealed intratumoral and intertumoral epithelium heterogeneity and tremendous differences between the tumor and normal epithelium. Epithelium cells and myeloid cells had more frequent cell‒cell communication than epithelium cells and T cells	[238]
	2021	Human	10 × Genomics	PRJNA777911	Uncovered heterogeneity in most cell types of the ESCC stroma, particularly in the fibroblast and immune cell compartments	[86]
Melanoma	2016	Human	Smart-seq2	DUOS-000002; GSE72056	Malignant cells within the same tumor displayed heterogeneity in the transcription of proteins related to the cell cycle, spatial context, and drug resistance program	[239]
	2020	Human	10 × Genomics	GSE139829	Analysis of tumor cells revealed previously unappreciated subclonal genomic complexity and transcriptional states	[94]
	2021	Human	10 × Genomics	GSE138665	Uncovered intratumoral heterogeneity at the genome and transcriptome level	[81]
Acute lymphoblastic leukemia	2020	Human	10 × Genomics	GSE132509	The predicted developmental states of cancer cells were inversely correlated with the expression levels of ribosomal protein, which could be a common contributor to intraindividual heterogeneity in childhood ALL patients	[240]
Diffuse large B cell lymphoma	2022	Human	10 × Genomics	CNGBdb: CNP0001940	High intratumor and intertumor heterogeneity was identified in DLBCL	[216]
	2022	Human	10 × Genomics	https://heidata.uniheidelberg.de	Provided an in-depth dissection of the transcriptional features of malignant B cells and the TME in DLBCL and new insights into DLBCL heterogeneity	[229]
Primary central nervous system lymphoma	2021	Human	10 × Genomics	GEO: GSE181304	Different subtypes of T cells and DCs showed significant heterogeneity	[85]
B-cell lymphoma	2020	Human	10 × Genomics	https://heidata.uni-heidelberg.de	Malignant subpopulations from the same patient responded strikingly differently to anticancer drugs ex vivo, highlighting the relevance of intratumor heterogeneity for personalized cancer therapy	[241]
Cutaneous T cell lymphoma	2018	Human	BD Precise assay	Correspondence with authors	Patients with SS displayed a high degree of single-cell heterogeneity within the malignant T-cell population, and the distinct subpopulation of malignant T cells exhibited HDACi resistance	[76]
	2019	Human	10 × Genomics	GSE128531	Provided an unprecedented view of lymphocyte heterogeneity and identifying tumor-specific molecular signatures, with important implications for diagnosis and personalized disease treatment	[77]
	2021	Human	10 × Genomics	GSE171811	Striking subclonal molecular heterogeneity was observed within clonal malignant T-cell populations in the skin and blood of patients with leukemic CTCL. The tissue microenvironment influenced the transcriptional state of malignant T cells, likely contributing to the evolution of malignant clones	[242]
	2022	Human	10 × Genomics	GSA-Human: HRA000166	Revealed the intratumor and interlesion diversity of CTCL patients, proposed a multistep tumor evolution model that further established a novel subtype, the T_CyEM_ group with a cytotoxic effector memory T-cell phenotype, and identified increased M2 macrophage infiltration	[78]
Subcutaneous panniculitis-like T cell lymphoma	2021	Human	10 × Genomics	GSA-Human: HRA000370	Provided insights into the heterogeneity of subcutaneous panniculitis-like T-cell lymphoma, as well as a better understanding of the transcription characteristics and immune microenvironment of this rare tumor	[208]

Buus et al. [76] used scRNA-seq and multicolor flow cytometry to analyze samples from 7 patients with Sézary syndrome (SS) and showed that these patients displayed a high degree of single-cell heterogeneity within the malignant T-cell population. Malignant T cells could be divided into distinct subpopulations based on heterogeneous surface marker expression and mRNA expression, and when treated with a histone deacetylase inhibitor (HDACi), some specific subpopulations were significantly reduced; however, the remaining subpopulations were largely unaffected. Gaydosik et al. [77] not only revealed intertumor T lymphocyte heterogeneity in cutaneous T-cell lymphoma (CTCL) skin tumors but also found that tumor-infiltrating CD8^+^ T lymphocytes exhibited heterogeneity in effector and exhaustion programs across patients, which provided an unprecedented view of lymphocyte heterogeneity in individual CTCL patients. Liu et al. [78] revealed intratumor and intertumor heterogeneity in the transcription and function of malignant T cells, and the activation/proliferation program profiles of malignant T cells in each patient identified with scRNA-seq analysis and TCR profiling were associated with the intratumor and intertumor heterogeneity of CTCL.

Heo et al. [79] employed scRNA-seq analysis in an in vitro model of ceritinib-resistant non-small cell lung cancer (NSCLC) to identify upregulation of cytidine deaminase (CDA) as a primary characteristic of anaplastic lymphoma kinase (ALK) inhibitor resistance. Additionally, the authors utilized single-cell Assay for Transposase-Accessible Chromatin using sequencing (scATAC-seq) to demonstrate that cells with acquired resistance may exhibit an open chromatin structure in the promoter and enhancer regions of *CDA*, potentially facilitated by DNA demethylation. Transcription factors such as TEAD1, SMAD3, and FOXM1 may be recruited to the regulatory region to induce overexpression of *CDA*, which promotes acquired resistance to ALK inhibitors. This study reveals the unexpected epigenetic heterogeneity and targeting CDA metabolism using epigenome-related nucleosides represents a potential new therapeutic strategy for overcoming ALK inhibitor resistance in NSCLC. Zhang et al. [80] performed a scRNA-seq analysis of tumor cells and identified five cell subgroups with distinct expression profiles in primary gastric adenocarcinoma (GAC). A panel of differentiation-related genes reveals a high diversity of differentiation degrees within and between tumors, and low differentiation degrees can predict poor prognosis in GAC, which offers valuable resources for deciphering gastric tumor heterogeneity and will provide assistance for precision diagnosis and prognosis.

In multiscale analyses using scRNA-seq of six different primary uveal melanomas, Pandiani et al. [81] uncovered an intratumoral heterogeneity at the genomic and transcriptomic levels. They deciphered a gene regulatory network underlying an invasive and poor prognosis state driven in part by the transcription factor HES6, which is a valid target to stop uveal melanoma progression. To dissect the cellular and molecular basis underlying hepatoblastoma (HB) oncogenesis and heterogeneity at the single-cell level, single-cell transcriptome profiling of tumor and paired distal liver tissue samples from five patients with hepatoblastoma was performed [82]. Seven distinct tumor cell subpopulations were annotated, and an effective three-level hepatoblastoma subtyping method was established based on their compositions. Facilitates chromatin transcription (FACT) inhibition could be a promising epigenetic-targeted therapeutic strategy against the CSC-like HB1-Pro-like1 subpopulation and its related high-risk “Pro-like1” subtype of HB.

In addition to the heterogeneity of tumor cells in cancers, nontumor cells also exhibit high heterogeneity. Presumably, tumor immune microenvironment (TIME) heterogeneity is largely derived from tumor heterogeneity and, in turn, influences cancer cell behaviors and clinical outcomes [83, 84]. Chen et al. [83] compared the TIME heterogeneity between gastric signet-ring cell carcinoma (GSRCC) and non-GSRCC by scRNA-seq. They found that compared to non-GSRCC, the GSRCC TIME appears to be quiescent, where Treg-FOXP3 and CD8-Tex are difficult to be mobilized, which further impairs the proper functions of B cells. Validated by the cytometry by time of flight (CyTOF) results, the decrease of CD8-Tex in GSRCC conflicted with the anticipation that the enrichment of this dysfunctional population would contribute to the worse prognosis of GSRCC. In a study of primary central nervous system lymphoma (PCNSL), different subtypes of T cells and dendritic cells (DCs) also showed significant heterogeneity [85]. Based on specific gene signatures, the T cells could be reclustered into four distinct subclusters, the T helper cell group, natural killer T (NKT)- cell group, MPC cell group, and classical T-cell group, and the DCs could be redivided into three subgroups: conventional dendritic cells (cDCs), myeloid dendritic cells (mDCs), and plasmacytoid dendritic cells (pDCs). Another scRNA-seq study about esophageal squamous cell carcinoma (ESCC) uncovered heterogeneity in most cell types of the ESCC stroma, particularly in the fibroblast and immune cell compartments. The authors revealed that tumor-specific *CSTl*^+^ myofibroblasts were associated with poor prognosis in ESCC [86]. To investigate the stromal heterogeneity of the TME in ovarian cancer, a research team used SRT to generate spatially resolved transcript profiles in treatment-naive advanced high-grade serous ovarian cancer (HGSOC) from long-term survivors (LTS) and short-term survivors (STS) [87]. They revealed high levels of intertumor and intratumor CAF heterogeneity, and novel spatially resolved CAF-tumor cross-talk signaling networks in the ovarian TME that are associated with LTS in patients with advanced HGSOC.

### Characterizing the TME

The TME, which comprises cellular and noncellular components, plays crucial roles in tumorigenesis, progression, invasion, metastasis, and drug resistance [7, 88]. Researchers have proposed that the TME might function as a double-edged sword in promoting or inhibiting tumor growth, which depends on the phase of tumor progression [89, 90]. Understanding the characteristics of the TME may help to understand the crosstalk between the TME and cancer cells and aid the development of novel strategies for tumor treatment [91]. Hallmark features of the TME include immune cells, stromal cells, blood vessels, and extracellular matrix. Among these components, immune cells are a critical factor in the TME and play a key role in tumorigenesis and treatment response [90, 92]. Characterizing the TME has attracted more and more researchers and many scRNA-seq studies have conducted in-depth studies of the TME (Table 3).

**Table 3:** Key findings related to characterizing TME among various tumors using scRNA-seq

Tumor	Year	Species	Protocol	Accession number (custom database if available)	Key findings	References
Lung cancer	2022	Human	10 × Genomics	https://doi.org/10.57760/sciencedb.02028	Identified a novel lymphocyte-related Mφ cluster named SELENOP-Mφ; a comprehensive depiction of the immune heterogeneity and definition of Mφ clusters could help design personalized treatment for lung cancer patients	[101]
Colorectal Cancer	2022	Human	DNBelab C4 platform	Correspondence with authors	Revealed significant differences in the TME between yCRC and oCRC; the TME of yCRC was more immunosuppressive than that of oCRC	[243]
	2022	Human	10 × Genomics	OEP001756	Generated a single-cell and spatial atlas of colorectal liver metastasis and found highly metabolically activated MRC1^+^ CCL18^+^ M2-like macrophages in metastatic sites. Efficient neoadjuvant chemotherapy can slow down such metabolic activation, suggesting that it may be possible to target metabolism pathways to prevent metastasis	[167]
Gastric cancer	2020	Human	10 × Genomics	Correspondence with authors	Identified different immune cell subtypes and their specific transcription factors. The IRF8 transcription factor was downregulated in CD8^+^ TILs from gastric cancer tissues compared to control tissues	[244]
	2020	Human	10 × Genomics	Correspondence with authors	Revealed widespread reprogramming across multiple cellular elements in the gastric cancer TME. Cellular remodeling was delineated by changes in cell numbers, transcriptional states, and intercellular interactions	[100]
	2023	Human	10 × Genomics	GSE212212	Provided a comprehensive molecular portrait of the immune cell composition and cell states in advanced gastric cancer patients, highlighting adaptive immune irresponsiveness in GSRCC and a mediator role of CXCL13 in the TIME	[83]
	2023	Human	10 × Genomics	HRA002108	Signature genes of PDGC were strongly enriched for the EMT program. DGC tended to be immune-rich type, whereas PDGC tended to be immune-poor (defined according to the density of tumor-infiltrating CD8^+^ T cells)	[143]
Liver cancer	2020	Human	10 × Genomics	CRA002308	Identified a subset of M2 macrophage with high expression of CCL18 and the transcription factor *CREM* that potentially participate in tumor progression and a new subset of activated CD8^+^ T cells highly expressing XCL1 that were correlated with better patient survival rates	[176]
	2022	Human	10 × Genomics	EGAC00001001616, GSE149614	Identified MMP9^+^ macrophages to be terminally differentiated TAMs and PPARγ to be the pivotal transcription factor driving their differentiation. Characterized heterogeneous subpopulations of malignant hepatocytes and their multifaceted functions in shaping the immune microenvironment of HCC	[177]
	2023	Human	10 × Genomics	Mendeley Data (skrx2fz79n)	Identified a TIB structure that was formed by the interaction of SPP1^+^ macrophages and CAFs, and this structure was related to immunotherapy efficacy. Blockade of SPP1 or macrophage-specific deletion of Spp1 in mice destroyed the TIB structure and sensitized HCC cells to immunotherapy	[102]
	2023	Human	10 × Genomics	PRJNA793914	TREM2^+^ TAMs played an important role in suppressing CD8^+^ T cells. TREM2 deficiency increased the therapeutic effect of anti-PD-L1 blockade by enhancing the antitumor activity of CD8^+^ T cells	[104]
Breast cancer	2018	Human	10 × Genomics	GSE113197	Revealed the existence of three distinct epithelial cell populations, one basal, and two luminal (secretory L1 and hormone-responsive L2) cell types, laying a foundation for understanding how the system goes awry during breast cancer	[245]
	2021	Human	10 × Genomics	HRA000477	Revealed significant heterogeneity in tumor-infiltrating B-cell subgroups and suggested local differentiation of infiltrating memory B cells within breast tumors	[97]
Esophageal cancer	2020	Human	10 × Genomics	GSE145370	Exhausted T and NK cells, Tregs, alternatively activated macrophages, and tolerogenic DCs were dominant in the TME. The crosstalk between macrophages and Tregs contributes to potential immunosuppression in the TME	[246]
	2021	Human	10 × Genomics	Correspondence with authors	Significant differences were found in stromal and immune cells between the esophagus normal and tumor tissues. LAG3 and HAVCR2 might be targets for immunotherapy in ESCC	[186]
	2022	Human	10 × Genomics	Correspondence with authors	Nonimmune stromal cells were significantly enriched in the TME. Most subsets of epithelial cells were enriched in the cancer regions, whereas inflammatory CAFs were enriched in the stromal regions	[247]
Ovarian cancer	2020	Human	Modified Smart-seq2	GSE146026	Significant interpatient variability in the composition and functional programs of ascites cells was found. Malignant cell variability was partly explained by differences in copy number alteration patterns and stemness program expression	[188]
	2022	Human	10 × Genomics	PRJNA784961; GSE189889	Acral melanoma had a suppressed immune environment compared with cutaneous melanoma from nonacral skin. Expression of multiple, therapeutically tractable immune checkpoints was observed, including PD-1, LAG-3, CTLA-4, VISTA, TIGIT, and ADORA2	[195]
Acute lymphoblastic leukemia	2021	Human	10 × Genomics	GSE172158	Two exhausted T-cell populations, characterized by upregulation of TIGIT, PDCD1, HLADRA, LAG3, and CTLA4, were discovered in B-ALL patients. Clonally expanded exhausted T cells were found to be likely to originate from CD8^+^ effector memory/terminal effector cells	[248]
Diffuse large B cell lymphoma	2021	Human	10 × Genomics	GEO: GSE182436	Resolved the DLBCL microenvironment at systems-level resolution and identified opportunities for therapeutic targeting	[214]
Follicular lymphoma	2019	Human	10 × Genomics	Correspondence with authors	Parallel measurement of single-cell expression in thousands of tumor cells and tumor-infiltrating lymphocytes can be used to obtain a systems-level view of the tumor microenvironment and identify new avenues for therapeutic development	[249]
	2022	Human	10 × Genomics	https://cellxgene.cziscience. com/collections/968834a0–1895–40df-8720–666029b3bbac	Follicular lymphoma microenvironment characteristics were associated with tumor cell mutations and MHC II expression	[250]
Angioimmunoblastic T cell lymphoma	2022	Human	10 × Genomics	Correspondence with authors	B cells within the AITL TME featured decreased expression of key markers such as CD73 and CXCR5. There was an expansion of distinct CD8^+^ T-cell populations with an exhausted phenotype and an expression profile indicative of dysfunction, impaired cytotoxicity, and upregulation of the chemokines XCL2 and XCL1	[251]
Classic Hodgkin lymphoma	2020	Human	10 × Genomics	EGA: EGAS00001004085	Provided detailed functional and spatial characteristics of immune cells in classic Hodgkin lymphoma at single-cell resolution. Identified a regulatory T-cell-like immunosuppressive subset of LAG3^+^ T cells contributing to the immune-escape phenotype	[93]

#### Immune cells

Of the immune cells, T cells are most studied by scRNA-seq analysis. According to gene expression signature profiling, diverse novel functional subgroups of classic T cells have been characterized, such as exhausted, cytotoxic, and immunosuppressive T cells. Aoki et al. [93] and Durante et al. [94] identified a novel regulatory T-cell-like immunosuppressive subset of lymphocyte activation gene 3 (LAG3)^+^ T cells that contribute to the immune-escape phenotype in classic Hodgkin lymphoma (CHL) and uveal melanoma (UM), respectively, that may be a target for immune checkpoint blockade (ICB). Kwon et al. [95] found that an increase in programmed death 1 (PD-1)^+^CD8^+^ T cells correlated with durable clinical benefit. In NSCLC, a special “preexhausted” T-cell cluster was successfully identified, and a high ratio of preexhausted to exhausted T cells was associated with a better prognosis [96]. In addition, the study also identified *TNFRSF9*^+^ regulatory T cells (Tregs) represented antigen-specific Tregs. Overall, tumor-infiltrating T cells might be much more complex than current knowledge suggests, and exploring functional T-cell clusters might provide new insight into the design of immunotherapy strategies.

Relatively speaking, B cells have been less studied than T cells. Hu et al. demonstrated that compared with those in peripheral blood, tumor-infiltrating B-cells have more mature and memory B-cell-like characteristics, higher clonality, a higher rate of class switch recombination, and more somatic hypermutations in breast cancer. Combined analyses suggested local differentiation of infiltrating memory B cells within breast tumors, and B-cell subgroups might contribute to immunosurveillance through various pathways [97].

Tumor-associated macrophages (TAMs), a specific subpopulation of macrophages, represent a large fraction of infiltrating immune cells within the TME in human cancers [98, 99]. An increasing number of scRNA-seq studies have found that macrophages are transcriptionally heterogeneous and do not conform to the traditional binary M1/M2 paradigm in many types of cancers [100–103]. Tan et al. revealed that TAMs expressed high levels of TREM2 in following transarterial chemoembolization (TACE), which played an important role in limiting the functions of CD8^+^ T cells and was associated with a worse clinical prognosis in hepatocellular carcinoma (HCC) [104]. You et al. [105] employed scRNA-seq to construct a single-cell atlas for a total of 23,010 individual cells from 6 patients with primary or recurrent malignant glioma and identified 5 cell types, including TAMs and malignant cells. M2-like TAMs were found to increase in recurrent malignant glioma significantly and the M2-like TAMs could activate the PI3K/Akt/HIF-1α/CA9 pathway in the malignant glioma cells via SPP1-CD44-mediated intercellular interaction.

Though granulocytes are a major component of the TME, their function in immunotherapy is still unclear. In an scRNA-seq study of CRC liver metastasis, IL-17 and the ferroptosis signaling pathway were significantly enriched in granulocytes, suggesting a potential role of the IL-17 signaling pathway in CRC liver metastasis. Abnormal ferroptosis-mediated cell death and Wnt signaling activation-induced neutrophil recruitment were proposed as causes of the higher levels of tumor-infiltrating granulocytes in CRC metastasis samples [106].

#### Stromal cells

Cancer-associated fibroblasts (CAFs) are a major component of the tumor stroma and play a critical role in facilitating crosstalk between cancer cells and the TME [92]. Increasing evidence has demonstrated that CAFs do not always exert a tumor-supportive role in oncogenesis, they may also play a tumor-suppressive effect that is context-dependent, namely phenotypic heterogeneity and functional diversity [107]. Many scRNA-seq studies have focused on the role of CAFs in tumor metastasis [108, 109], and their effect on prognosis [86, 110], and CAFs may be potential therapeutic targets. Li et al. [98] found that inflammatory CAFs (iCAFs) and extracellular matrix CAFs (eCAFs) not only exhibited enhanced pro-invasive activities but also mobilized the surrounding immune cells to construct a tumor-favorable microenvironment in gastric cancer. In particular, eCAFs were associated with a shorter overall survival (OS) time of patients with gastric cancer. Another study revealed that matrix cancer-associated fibroblasts (mCAFs) expressing α-SMA, vimentin, COL3A, COL10A, and MMP11 could enhance cancer cell invasion in HGSOC [97]. In another scRNA study, several types of stromal cells were identified in high-grade serous tubo-ovarian cancer (HGSTOC). The study showed that the high relative frequency of myofibroblasts, TGF-β-driven CAFs, mesothelial cells, and lymphatic endothelial cells could predict poor outcomes, while high levels of plasma cells correlated with more favorable outcomes [100]. Guo et al. [101] used multimodal intersection analysis (MIA) to integrate scRNA-seq and SRT, and the exact cellular components of the tumor and stromal regions were annotated in the three ESCC samples. The results indicated that the various stromal cell subpopulations were heterogeneous. Compared with immune cells, non-immune stromal cells were significantly enriched in the TME. Most subsets of epithelial cells were enriched in the cancer regions, while inflammatory CAFs were correlated with the stromal regions.

Nonhematopoietic cells (NHCs) are closely correlated with B cells in neoplastic follicles and play a major role in supporting follicular lymphoma (FL) [111]. Abe et al. [112] constructed a single-cell transcriptome atlas of more than 100,000 NHCs collected from 27 human samples, including 10 FL samples, and revealed 30 distinct subclusters, including some that were previously unrecognized. The 30 subclusters were composed of 10 subclusters of blood endothelial cells (BECs), 8 subclusters of lymphatic endothelial cells (LECs), and 12 subclusters of nonendothelial stromal cells (NESCs). This study identified that human lymph nodes (LNs) harbor unique NHC subpopulations that have not been detected in murine LNs. The authors observed the remodeling of NHC proportions in FL. The proportion of BECs was markedly increased in FL relative to metastasis-free LNs (MFLNs), whereas the proportion of LECs was decreased. Moreover, the proportion of arterial subclusters was increased in FL BECs. In FL NESCs, the proportion of follicular dendritic cells (FDCs) was substantially increased. Notably, the proportion of marginal reticular cells (MRCs) was also greatly increased in FL, whereas the proportions of adventitia stromal cells (SCs), SFRP4-SCs, SFRP2-SCs, and TNF-SCs were decreased.

Somatic mutations as well as somatic copy number alterations (SCNAs) are found in normal colorectal epithelial cells by whole-genome bulk sequencing of normal colorectal crypts and are considered to be a precancerous phenomenon [113]. However, few studies have been published on SCNAs of TME cells. Zhou et al. [114] performed scRNA-seq-plus-genomics of 21 patients with microsatellite-stable CRCs and 6 cancer-free, elderly individuals. SCNAs are prevalent in immune cells, fibroblasts, and endothelial cells in both the TME and the normal tissues of each individual. Moreover, the proportions of fibroblasts with SCNAs in tumors are much higher than those in adjacent normal tissues.

### CTCs

Circulating tumor cells (CTCs) are vital components of liquid biopsies for the diagnosis of residual cancer, monitoring of therapy response, and prediction of recurrence [115]. Transcriptomics of CTCs represents an attractive opportunity to bridge the knowledge gap and develop novel biomarkers, and analysis of CTCs collected from patient blood may provide a new perspective for understanding the drug resistance of tumors and reveal a broad range of targets for use in the field of precision oncology [116–118]. Kozuka et al. [119] conducted a study in which CTCs were collected from metastatic colorectal cancer (mCRC) patients without relying on any traditional CTC markers, such as epithelial and mesenchymal cell antigens, and were subjected to scRNA-seq using SMART-seq v4. The results showed that mCRC patients receiving second or later-line treatment who had epithelial–mesenchymal transition (EMT) gene-expressing CTCs had significantly shorter progression-free survival (PFS) and OS. Another scRNA-seq study also proposed that CTC enumeration and scRNA-seq analysis might predict response to therapy in the treatment of melanoma patients [120].

### CSCs and LSCs

Cancer stem cells (CSCs) have a slow growth rate and are resistant to chemotherapy and radiotherapy which lead to the failure of traditional current therapy and have been recognized as promising therapeutic targets for cancer therapy [121, 122]. Zheng et al. found that distinct genes within different CSC subpopulations were independently associated with HCC prognosis, suggesting that the diverse hepatic CSC transcriptome is related to intratumor heterogeneity and tumor progression [123]. Another scRNA-seq study revealed that Yes-associated protein 1 (YAP1) was highly upregulated in peritoneal carcinomatosis (PC) tumor cells, conferred CSC properties, and appeared to function as a metastasis driver. Pharmacologic inhibition of YAP1 specifically reduced CSC-like properties and suppressed tumor growth in YAP1^high^ PC cells, especially in combination with cytotoxic agents in an in vivo patient-derived xenograft (PDX) model [124].

The inevitable chemotherapy resistance and high relapse rate of acute myeloid leukemia (AML) are mainly caused by the persistence of leukemia stem cells (LSCs). [125]. The identification of the main features of LSCs may improve diagnosis and treatment [126]. Naldini et al. [127] discriminated LSCs from regenerating hematopoietic cells and assessed their longitudinal response to chemotherapy by detecting nucleophosmin 1 (*NPM1*) mutation or chromosomal monosomy by single-cell transcriptomic analyses. The researchers provided evidence for a classical LSC model in NPM1^mut^ AML, where nonresponse/relapse was strongly correlated with a high proportion of quiescent miR-126^high^ LSCs at diagnosis. This research provides a framework for stratifying patients based on the presence of miR-126^high^ LSC scRNA-seq data and to identify therapeutic targets for LSC eradication.

### Tumorigenesis and clonal evolution

Tumorigenesis and cancer progression are multistage, complex, and dynamic evolutionary processes, that result from diverse gene changes [128]. Genes or cell subclusters that play crucial roles in tumorigenesis and the development of tumors have been identified using single-cell sequencing [91, 129]. By performing an scRNA-seq analysis of individual patients and potential heat diffusion for affinity-based trajectory embedding (PHATE) analysis in combination with scTCRseq and CTCL clonotyping, Ren et al. [130] identified putative precancerous circulating populations characterized by an intermediate stage of gene expression and a mutation level between that of normal CD4^+^ T cells and malignant CTCL cells. Song et al. [131] performed scRNA-seq on 16 transformed CTCL (tCTCL) skin biopsies and identified a core oncogenic program that malignant T cells exploited to acquire aggressive behaviors and a survival advantage with transformation. Oxidative phosphorylation (OXPHOS) and MYC were the top enriched pathways, and their activity progressively increased as the disease evolved, followed by EMT/stemness and E2F target genes; downregulation of MHC I was suggestive of immune escape.

Recent genetic and epigenetic studies of the disease course of leukemia and other hematological neoplasias have provided important insights into the role of clonal evolution as a driver of tumor initiation, disease progression, and relapse [132]. Clonal mutations are shared by all cancer cells, whereas subclonal mutations are present only in a subset [133]. An scRNA-seq study revealed marked expansion of abnormal germinal center B-cell-like (GCB)-related clusters simultaneously exhibiting a cell activation profile like that of light zone (LZ)-GCB cells and a cell proliferation profile like that of dark zone (DZ)-GCB cells in both mouse and human angioimmunoblastic T-cell lymphoma (AITL) samples [134]. These age-related clonal hematopoiesis (ACH)-derived GCB cells harboring *TET2* mutations can independently undergo clonal evolution and function as microenvironmental cells to support AITL tumorigenesis. Tan et al. [135] cross-analyzed healthy donors, asymptomatic carriers, and patients with adult T-cell leukemia/lymphoma (ATLL) using scRNA-seq and T-cell receptor (TCR)-seq to determine the seamless transition of naive T cells into activated T cells and fully understand how HTLV-1 infection controls physiological pathways in T cells and transforms them into ATLL cells; HTLV-1-infected cells in an activated state further transformed into ATLL cells, which were characterized as clonally expanded, highly activated T cells. The expression of HLA class II genes in HTLV-1-infected cells was uniquely induced by the viral protein Tax and further upregulated in ATLL cells. Functional assays revealed that HTLV-1-infected cells upregulated HLA class II molecule expression and acted as tolerogenic antigen-presenting cells to induce anergy of antigen-specific T cells.

### Cell‒cell interactions

Tumors are complex ecosystems defined by the interaction between heterogeneous cell types (including malignant, immune, and stromal cells) that communicate by ligand‒receptor interactions, which may play a key role in the development of cancer and maybe therapy targets [136, 137]. Cell‒cell interactions focus on interactions between malignant cells and the TME or cells from the TME [78, 138]. In addition to interactions between tumor cells and stromal cells, interactions among stromal cells have also received attention. Abe et al. [112] found that medullary and adventitial stromal cells had significant interactions with malignant B cells through CD70-CD27 interaction in FL and proposed stroma-derived CD70 as a potential biomarker and therapeutic target for FL. In gastric cancer samples, Li et al. [139] observed enhanced interactions between endothelial cells and multiple cell types, including fibroblasts, monocytes, macrophages, and DCs. The strong interaction between endothelial cells and fibroblasts implies that fibroblasts are closely related to tumor angiogenesis and maintenance of the tumor vasculature.

### Immunosurveillance and immune evasion

Immune evasion is a hallmark of cancer [140]. According to the immunosurveillance theory, neoplastic cells can progress to generate clinically obvious cancer only if they escape from the control of immunological effector cells [141]. One study leveraged scRNA-seq data from 33 melanoma tumors and computational analyses to reveal malignant cell states that promote immune evasion. The researchers identified a resistance program expressed by malignant cells that was associated with T-cell exclusion and immune evasion. The program was expressed prior to immunotherapy and was enhanced following immunotherapy in resistant lesions, and the expression of this program could predict clinical responses to anti-PD-1 therapy in melanoma patients [142]. Zhou et al. [143] performed scRNA-seq on normal mucosa tissue, differentiated gastric cancer (DGC) tissue, poorly differentiated gastric cancer (PDGC) tissue, and neuroendocrine carcinoma (NEC) tissue from gastric cancer patients. Interestingly, they found that along the trans-differentiation path from DGC to NEC, immune evasion was gradually increased with decreasing interferon pathway response activity in malignant cells, though this finding needs further functional investigation.

### Metabolic reprogramming

Metabolic reprogramming, also known as deregulated cellular metabolism, is one of the emerging hallmarks of cancer that occurs as a result of the metabolic plasticity of cancer cells [144]. Metabolic reprogramming in tumor cells is dynamic and variable, dependent on the tumor type and microenvironment, and reprogramming involves multiple metabolic pathways, which is considered a promising therapeutic target against tumors [145, 146]. Fernández-García et al. [147] used scRNA-seq to define the metabolic reprogramming of CD8^+^ T cells that were becoming activated and/or differentiating. They identified a differential time-dependent reliance of activating T cells on the synthesis versus the uptake of various nonessential amino acids. Further research proposed that the expression of ASNS affects the outcome of CD8^+^ T-cell differentiation and that ASNS overexpression enhances CD8^+^ T-cell effector function and antitumor responses. Xu et al. [148] established a systematic landscape of metabolic heterogeneity and its relationship with immunity in the AML microenvironment at single-cell resolution for the first time. They focused on the metabolic preference of AML progenitor cells and diverse immune cells and proposed potential targets for AML metabolic therapy, including ENO1, GSTP1, MT-ND4L and UQCR11.

### Transcription factors and transcriptional programs

Dysregulation of transcription factor activity unsurprisingly drives tumorigenesis and oncogenic transformation [149]. Targeting transcription is a highly promising anticancer strategy [150]. Before a transcription factor can become a bona fide drug target, the underlying biological properties of that protein must be understood [151]. Rastogi et al. [152] demonstrated that nuclear factor I-C (NFIC) overexpressing monocytes had increased expression of growth and survival genes. NFIC knockdown in an ex vivo mouse MLL::AF9 preleukemic stem cell model decreased the growth and colony formation of the cells and increased their expression of the myeloid differentiation markers Gr1 and Mac1. These results indicate that NFIC is an important transcription factor involved in myeloid differentiation as well as AML cell survival and is a potential therapeutic target in AML.

Transcription factors do not generally function alone and rather cooperate to control gene expression [153]. Sun et al. [154] comprehensively mapped malignancy-related transcription factor regulatory networks activated in different AML subtypes by analyzing scRNA-seq data from AML patients and healthy donors. They identified six modules of regulatory networks that were prevalently dysregulated in all AML patients. AML subtypes featuring different malignant cell compositions possessed subtype-specific regulatory transcription factors associated with suppression of differentiation or immune modulation. Collectively, this study thoroughly revealed the abnormal spectrum of transcriptional regulatory networks in AML and revealed that dysregulation was subtype-specific, providing insights into AML pathogenesis and potential targets for both diagnosis and therapy.

## Clinical applications of scRNA-seq in solid tumors

Solid tumors account for the vast majority of cancer incidence and death. ScRNA-seq has been used in the study and application of various solid tumors (Table 4).

**Table 4:** Clinical significance of scRNA-seq in various solid tumors

Tumor	Year	Species	Protocol	Accession number (custom database if available)	Clinical significance	References
Lung cancer	2021	Human	10 × Genomics	Correspondence with authors	Provided single-cell transcriptomic profiles of SSNs and their TME that helped advance lung cancer immunotherapy	[252]
	2022	Mouse	10 × Genomics	GSE180963; GSE182228	ICAM1 on tumor cells orchestrates antitumor immune response, especially in adaptive immunity	[159]
	2023	Human	BD Rhapsody Single-Cell Analysis System	HRA001033	Neoadjuvant PD-1 blockade combined with chemotherapy was associated with the emergence of distinct NSCLC tumor microenvironment transcriptomes that correlated with therapy response	[160]
Colorectal Cancer	2020	Human	Smart-seq2	PRJEB34105; GSE146771	Two distinct TAM subsets showed inflammatory and angiogenic signatures, and showed differential sensitivity to CSF1R blockade. Anti-CD40 therapy activated specific cDC1s and expanded Th1-like and CD8^+^ memory T cells	[163]
	2020	Human	STRT-seq	HRA000201	SCNAs were prevalent in immune cells, fibroblasts, and endothelial cells in the TME of CRC, and the proportion of SCNA fibroblasts in tumor was much higher than that in normal tissue. There was clonal expansion of fibroblasts with SCNAs in the tumor, especially the tumors with chr7 gain	[114]
	2021	Mouse	10 × Genomics	SUB8983993	The intratumoral immunomodulation induced by CD73 inhibition is distinct from that induced by PD-1 inhibition, and agents inhibiting CD73 have potential as novel anticancer immunotherapies for CRC that may have synergistic effects when combined with PD-1 blockade treatments	[166]
	2021	Human	SMART seq v4	PRJNA759644	Identified 59 single CTCs which were classified into four groups based on EMT and stem cell-related gene expression. Patients receiving second or later-line treatment who had CTCs expressing EMT genes had significantly shorter PFS and OS	[119]
	2021	Human	10 × Genomics	Correspondence with authors	Tumor mutational burden was associated with distinct immune profile patterns in human CRC. Identified phenotypic and functional diversity of tumor-associated macrophages and T cells	[253]
	2022	Human	Modified STRT-seq protocol and SMART-seq2	HRA000183	Provided insights into how driver mutations interfere with the transcriptomic state of cancer cells in vivo at a single-cell resolution knowledge on metastatic mechanisms as well as potential markers and therapeutic targets for CRC diagnosis and therapy	[162]
Gastric cancer	2021	Human	Smart-seq2	GSE158631	Discovered gastric cancer lymph node metastasis marker genes (*ERBB2*, *CLDN11,* and *CDK12*), as well as potential gastric cancer evolution-driving genes (*FOS* and *JUN*)	[168]
	2022	Human	10 × Genomics	Correspondence with authors	PD-1 expression in CD8^+^ T cells might predict clinical responses to PD-1 blockade therapy in gastric cancer	[254]
	2022	Human	10 × Genomics	HRA002336; GSE206785	Revealed 81 well-defined TME cell types from nonepithelial origin, and these cell types together with immunosuppressive myeloid cell subsets and regulatory T cells established an immunosuppressive microenvironment that correlated with worse prognosis and lack of response in anti-PD1-treated patients	[171]
	2022	Human	10 × Genomics	GSE183904	Identified increased plasma cell proportions as a novel feature of diffuse-type tumors, and uncovered distinct CAF subtypes with INHBA-FAP-high cell populations as predictors of poor clinical prognosis	[110]
	2022	Human	10 × Genomics	Correspondence with authors	iCAFs and eCAFs not only exhibited enhanced pro-invasive activities but also mobilized surrounding immune cells to construct a tumor-favorable microenvironment	[109]
	2022	Human	10 × Genomics	PRJCA002596	Tregs were significantly enriched in gastric tumor tissues with increased expression of immune suppression related genes. *ACKR1* was specifically expressed in tumor endothelial cells, and high *ACKR1* expression was associated with poor prognosis in the cohort data, potentially revealing a novel target for gastric cancer treatment	[139]
	2022	Human	10 × Genomics	PRJNA776683	Abnormal neutrophil polarization and maturation and activation of the immune checkpoint SPP1 might contribute to LN metastasis in gastric cancer	[169]
Liver cancer	2018	Human	10 × Genomics	GSE103591	Different CSC subpopulations had distinct molecular signatures, and distinct genes within different CSC subpopulations were independently associated with HCC prognosis	[123]
	2022	Human	10 × Genomics	EGAS00001004843	Identified early predictors of clinical response to anti-PD-1 ICB in patients with HCC, and proposed a new combination immunotherapy of anti-PD-1 and anti-TNFR2 for HCC that may enhance response without exacerbating irAEs	[255]
	2022	Mouse	10 × Genomics	GSE212047	The dynamic shift in HSC subpopulations and their mediators during chronic liver disease is associated with a switch from protection against HCC to HCC promotion	[173]
	2022	Human	BD Rhapsody platform	Correspondence with authors	Revealed B-cell-driven type 2 innate inflammation and a potential strategy for HCC immunotherapy	[174]
	2022	Human	10 × Genomics	OEP002779	FACT inhibition was identified as a promising epigenetic-targeted therapeutic strategy	[82]
	2022	Mouse	10 × Genomics	GSE181515	Depletion of Prom1^+^ cells impeded tumor growth and reduced cancer hallmarks. The Prom1-lineage gene signature predicted poor prognosis in HCC, and enrichment of reactive oxygen species detoxification genes was key for lineage propagation	[178]
	2023	Mouse	10 × Genomics	PRJNA825069	Single anti-PD-1 treatment appears to be effective in HCCs with genetic mutations driving hot tumors, while combined anti-PD-1 and sorafenib treatment may be more appropriate in HCCs with genetic mutations driving cold tumors	[175]
Breast cancer	2020	PDX models	SmartSeq2	GSE123837	Pharmacological inhibition of OXPHOS dramatically attenuated metastatic seeding in the lungs, revealing the potential of targeting OXPHOS to prevent metastatic spread in patients with breast cancer	[74]
	2021	Human	10 × Genomics	Correspondence with authors	Basal-like breast cancer (ER^neg^) might originate from luminal progenitors, and ER^high^ luminal breast cancer might originate from mature luminal cells in *BRCA1* mutation carriers	[180]
	2021	Mouse	Fluidigm C1 platform	GSE148614	BRCA1 deficient tumors could be classified into four subtypes with distinct molecular features and different sensitivities to anticancer drugs at the intertumor level and reconstructed the BRCA1 related mammary tumorigenesis to uncover transcriptomes alterations	[181]
	2023	Human and mouse	10 × Genomics	GSE190772; GSE210286	The combination of a p38i, anti-OX40 therapy, and cytotoxic T-cell engagement cured mice of metastatic disease and produced long-term immunologic memory	[182]
Esophageal cancer	2021	Human	10 × Genomics	PRJNA777911	Identified a tumor-specific subset of CST1^+^ myofibroblasts with prognostic value, potential biological significance, and cancer-specific expression of immune checkpoint inhibitors	[86]
	2021	Human	10 × Genomics	GSE160269	The expression levels of the identified 14 genes were significantly associated with survival time in ESCC patients	[187]
Ovarian cancer	2021	Human	10 × Genomics	GSE147082	TOX-expressing CD8^+^ Trm and granulysin-expressing CD4^+^ T-cell clusters were enriched in the high T_inf_ group. The high T_inf_ group had an antitumor response	[256]
	2021	Human	10 × Genomics	EGAS00001004987	High relative frequencies of myofibroblasts, TGF-β-driven CAFs, mesothelial cells, and lymphatic endothelial cells predicted poor outcomes, while a high frequency of plasma cells correlated with a more favorable outcome	[192]
	2022	Human	10 × Genomics	PRJCA009148	Expression of the ERBB2 and HOXB-AS3 genes was higher in metastatic tumors than in primary tumors. CAFs with EMT-program were enriched in HG_M	[189]
	2022	Human	10 × Genomics	DRA012826; DRA012827	A cancer-initiating cell population and a population expressing CA125 survived initial treatment, suppressed antitumor immunity, and were associated with poor prognosis	[193]
	2022	Human	10 × Genomics	GSE184880	Identification of a model including four EMT-related genes for the prediction of HGSOC patient outcomes, the role of mCAFs in enhancing ovarian cancer cell invasion, and the potential therapeutic value of anti-TIGIT treatment	[108]
	2022	Human	10 × Genomics	EGAS00001005010	Identification of a consistent increase in the stress-associated cell state during chemotherapy. The stress-associated state existed before chemotherapy, was subclonally enriched during the treatment, and was associated with poor PFS	[257]
Melanoma	2018	Human	Smart-seq2	GSE115978	Identified a resistance program expressed by malignant cells that was associated with T-cell exclusion and immune evasion. CDK4/6 inhibitors repressed the program and might sensitize melanoma to immunotherapy	[142]
	2018	Human	Smart-seq2	phs001680.v1.p1; GSE120575	Revealed distinct CD45^+^ cells associated with clinical outcome. The balance between two CD8^+^ T-cell states was linked with tumor regression. TCF7^+^CD8^+^ T-cell frequency in tumor tissue predicted response and better survival. Dual blockade of CD39 and different checkpoint proteins enhanced immunity	[258]
	2020	Human	10 × Genomics	GSE139829	LAG3 was identified as a potential candidate for immune checkpoint blockade in patients with high-risk UM	[94]
	2021	Human	10 × Genomics	GSE138665	Revealed a gene regulatory network underlying an invasive state and poor prognosis driven in part by the transcription factor HES6. HES6 was identified as a valid target to stop uveal melanoma progression	[81]
	2021	Human	10 × Genomics	Correspondence with authors	Provided the first atlas of two distinct sites of melanoma CNS metastases and defined the immune cell landscape. Identified the presence of a rare population of DCs that was associated with increased OS	[259]
	2021	Human	10 × Genomics	phs002944.v1.p1; GSE185386	Chromosomal instability is associated with brain metastasis. Cancer cells in the brain metastases were enriched for a neuronal-like metaprogram. Macrophages had a pro-tumorigenic phenotype in brain metastases	[260]
	2022	Human	Smart-seq2	Correspondence with authors	Melanoma CTCs could be served as biomarkers for disease monitoring	[120]
	2022	Mouse	10 × Genomics	GSE211602	ASNS overexpression enhanced CD8^+^ T-cell effector function and antitumor responses	[147]
	2023	Human	10 × Genomics	EGAS00001005580	The highest LAG3 expression was noted in NK cells, Tregs, and CD8^+^ T cells, and these cell populations underwent the most significant changes during treatment. LAG3^+^ Tregs expanded but, based on their transcriptome profile, became metabolically silent during the treatment	[194]

### Lung cancer

#### Cancer metastasis

Previous scRNA-seq studies related to lung cancer have been limited to early-stage primary tumors and normal tissues resected from a small number of samples of mixed histological types [96, 155]. In a recent scRNA-seq study, the authors identified a cancer cell subtype that deviated from the normal differentiation trajectory from 208,506 cells populating the normal tissues and early to metastatic cancer tissues of 44 patients, and this subtype was specifically associated with cancer progression and metastasis in lung adenocarcinoma (LUAD) patients [156]. Analysis of stromal and immune cell dynamics revealed ontological and functional changes that created a protumoral and immunosuppressive microenvironment. Normal resident myeloid cell populations were gradually replaced with monocyte-derived macrophages and DCs, accompanied by T-cell exhaustion.

#### Disease monitoring

Obtaining high-quality samples of metastatic human tumors, particularly at multiple treatment time points, is difficult. A paucity of previous single-cell studies that sample metastatic malignancies and prior scRNA-seq studies of metastatic disease only focused on single treatment time points or before treatment [155, 156]. Maynard et al. [157] performed scRNA-seq on 49 clinical biopsies obtained from 30 patients before and during targeted therapy. The results revealed that cancer cells surviving therapy in residual disease (RD) samples expressed an alveolar-regenerative cell signature suggesting a therapy-induced primitive cell-state transition, whereas those present in progressive disease (PD) samples had upregulated kynurenine, plasminogen, and gap-junction pathways. Active T-lymphocytes and decreased macrophages were present in RD samples, and immunosuppressive cell states characterized PD samples. This research provided a foundation to develop strategies for the elimination or neutralization of RD to induce more durable responses for patients with advanced-stage NSCLC and potentially other solid malignancies treated with various therapeutic modalities.

#### Treatment

The molecular heterogeneity of NSCLC has not been comprehensively analyzed. Li et al. [158] performed high-precision scRNA-seq analyses on 7364 individual cells from tumor tissues and matched normal tissues from 19 primary lung cancer patients and 1 pulmonary chondroid hamartoma patient. They identified a significant proportion of cancer cells simultaneously expressing classical marker genes for two or even three histologic subtypes of NSCLC—adenocarcinoma (ADC), squamous cell carcinoma (SCC), and neuroendocrine tumor (NET). These cells were defined as mixed-lineage tumor cells, and genes specific to mixed-lineage tumor cells were identified, including *AKR1B1.* Further experiments showed that gene knockdown and small molecule inhibition of *AKR1B1* significantly decreased cell proliferation and promoted cell apoptosis, suggesting that *AKR1B1* plays an important role in tumorigenesis and could be a target for tumor therapy in NSCLC patients with mixed-lineage tumor features. A previous scRNA-seq study only characterized the T-cell landscape and neglected the dynamics and molecular features of the immune landscape in lung cancer at single-cell resolution [96]. Wang et al. [101] performed scRNA-seq on 72,475 immune cells from 40 samples of tumor and matched adjacent normal tissues from19 NSCLC patients and identified a novel lymphocyte-related subcluster named *SELENOP*-macrophages (Mφ), which highly expressed *FOLR2*, *IL32*, *CD3D*, and *LTC4S*. Survival analyses based on established TCGA data showed that the *SELENOP*-Mφ cluster might play an antitumor role in LUAD. Another scRNA-seq study revealed that ectopic expression of intercellular adhesion molecule-1 (ICAM1) in liver kinase B1 (*LKB1*) -deficient tumors increases the homing and activation of adoptively transferred SIINFEKL-specific CD8^+^ T cells, reactivates tumor-effector cell interactions and resensitizes tumors to ICB [159]. The results revealed that ICAM1 on tumor cells orchestrates the antitumor immune response, especially in adaptive immunity.

#### Drug resistance

Most patients are refractory to immunotherapy or acquiring resistance. Hu et al. [160] characterized the transcriptomes of ~ 92,000 single cells from 3 pretreatment and 12 posttreatment samples from patients with NSCLC who received neoadjuvant PD-1 blockade combined with chemotherapy. They identified increased serum estradiol and two cell types in the TME (FCRL4^+^FCRL5^+^ memory B cells and CD16^+^CX3CR1^+^ monocytes) that could serve as biomarkers for a “positive feedback” immune response and a “negative feedback” response, respectively.

### Colorectal cancer

#### Tumorigenesis and metastasis

Metastasis of CRC remains a major problem after curative treatment and is an important cause of CRC-related death [161]. Wang et al. [162] performed whole genome sequencing (WGS), multiregion whole exome sequencing (WES), simultaneous scRNA-seq, and single-cell targeted cDNA Sanger sequencing on matched adjacent normal, primary tumor, and metastatic tumor tissues from 12 mCRC patients. The results indicated that aberrant activation of the PPAR signaling pathway plays a critical role in CRC tumorigenesis. By analyzing matched samples from the same patient, distinct origins of tumors that had metastasized to the lymph nodes versus the liver were revealed, which somewhat contradicts with traditional ideas that distant organ metastasis is seeded through the lymph nodes. These findings offer novel insights regarding metastasis mechanisms as well as potential markers and therapeutic targets for CRC diagnosis and therapy.

#### Treatment

Few studies have applied scRNA-seq to dissect the mechanisms underlying immune-modulating therapies. Zhang et al. [163] performed scRNA-seq analyses on immune and stromal populations from CRC patients. Treatment with anti-CSF1R monotherapy preferentially depleted *C1QC*^+^ TAMs with an inflammatory signature but spared *SPP1*^+^ TAMs expressed pro-angiogenic/tumorigenic genes in mice and humans, and specific depletion of *SPP1*^+^ TAMs might ultimately lead to improved outcomes of myeloid-targeted immunotherapy or enhance ICB combination therapies. In addition, treatment with a CD40 agonist antibody preferentially activated the *Ccl22*^+^ cDC population and increased *Bhlhe40*^+^ T helper 1 (Th1)-like cells and CD8^+^ memory T cells. The previous studies found that the *BHLHE40* + Th1-like cell population is significantly enriched in tumor samples from CRC patients with high microsatellite instability (MSI), who respond to ICB [164, 165]. Another scRNA-seq study of mice determined that AB680, a selective inhibitor of the CD73 ectoenzyme improved the anticancer functions of immunosuppressive cells such as Tregs and exhausted T cells, while PD-1 blockade reduced the number of *Malat1*^high^ Tregs and M2 macrophages [166]. Their intratumoral immunomodulation was distinct, and AB680 might be a novel treatment for patients with refractory CRC who do not respond to existing anticancer chemotherapy drugs and PD-1 antagonists. Wu et al. [167] sequenced 97 matched samples using scRNA-seq and spatial transcriptomics analysis, and found that suppressive *MRC1*^+^*CCL18*^+^ macrophages displayed the highest metabolic activity and underwent remarkable spatial reprogramming. Neoadjuvant chemotherapy (NAC) could block this activity and restore the antitumor immune balance in responsive patients, whereas nonresponsive patients showed a more suppressive state.

### Gastric cancer

#### *Cancermetastasis*

The mechanism of gastric cancer lymph node metastasis remains unknown, partly because data from metastasis studies were generated with the bulk approach, which was likely to mask the roles of subpopulations. Wang et al. [168] performed scRNA-seq on samples from the primary tumors and metastatic lymph nodes (MLNs) of three gastric cancer patients. The authors found a subgroup of cells between the metastatic group and primary group and discovered some gastric cancer lymph node metastasis marker genes (*ERBB2*, *CLDN11*, and *CDK12*), as well as potential gastric cancer evolution-driving genes (*FOS* and *JUN*). Another scRNA-seq study of gastric cancer organ-specific metastasis (liver, peritoneum, ovary, lymph node) revealed that immune and stromal cells exhibited cellular heterogeneity and created a protumor and immunosuppressive microenvironment. In addition, a 20-gene signature of LN-derived exhausted CD8^+^ T cells might predict LN metastasis. Recently, Qian et al. [169] performed scRNA-seq on tissues from primary tumors and MLNs of gastric cancer patients to explore the differences in tumor cells and the TME between gastric cancer primary tumors and MLNs. The authors identified a malignant subpopulation showing the potential for LN metastasis, which displayed high translation initiation and protein activity. In addition to malignant cells, abnormal neutrophil polarization and maturation and activation of the immune checkpoint SPP1 might contribute to LN metastasis of gastric cancer.

#### Disease monitoring

Kwon and colleagues used WES and scRNA-seq on serial and multi-region tissue samples in addition to serial peripheral blood analyses with samples from advanced microsatellite instability-high (MSI-H) gastric cancer patients in a phase II trial of pembrolizumab [95]. The results supported clear differences in both baseline and adaptive TME composition between responders and nonresponders. Nonresponders had frequent mutations and upregulation of the Wnt/β-catenin pathway and increased CAF abundance. It is interesting to note that decreased T-cell infiltration and lower NK-cell numbers were observed in nonresponders.

#### Treatment

Early-stage gastric cancer is mainly treated with surgery, while for advanced gastric cancer, the current treatment options remain insufficient [170]. Li et al. [139] performed scRNA-seq on nine untreated nonmetastatic gastric cancer patients and found that *ACKR1* was specifically expressed in tumor endothelial cells. This gene was associated with poor prognosis in the cohort data and was thus reported to be a potential novel target for gastric cancer treatment. Another study found that activation of the SPP1-CD44 interaction in MLNs was related to the suppression of T-cell activation in the MLN, which might be a therapeutic target in gastric cancer patients with lymph node metastasis [169]. Moreover, selective inhibitors of the Wnt/β-catenin pathway could be promising in combination with immune checkpoint inhibitors (ICIs) in gastric cancer [95].

#### Prognosis

ITH is a fundamental property of cancer; however, the origins of ITH remain poorly understood. Wang et al. [72] performed scRNA-seq of PC samples from 15 patients with GAC, explored the ITH of malignant PC cells and identified factors significantly correlated with patient survival. Single-cell analysis of ITH was used to classify PC specimens into two subtypes: gastric-dominant (mainly gastric cell lineages) and GI-mixed (with mixed gastric and colorectal-like cells), and both had prognostic values independent of clinical variables. Further analyses found that patients with GI-mixed molecular features in their PC tumor cells survived significantly longer than those with gastric-dominant features probably because of intestinal metaplasia. In addition, all patients whose tumors had 17q gain were short-term survivors. Last, the authors discovered a 12-gene signature that appeared to be fundamental to GAC carcinogenesis/progression as it was not only highly prognostic in the GAC-PC validation cohort but performed just as robustly in several large-scale localized GAC cohorts. Kang et al. revealed that activated fibroblasts and endothelial cells together with immunosuppressive myeloid cells and Tregs established an immunosuppressive microenvironment that correlated with worse prognosis and lack of response in anti–PD-1-treated patients. In contrast, a subset of IFNγ activated T cells and HLA-II expressing macrophages was found to be linked to treatment response and increased OS [171]. Kumar et al. generated a comprehensive single-cell atlas of gastric cancer (> 200,000 cells) based on data from 48 samples from 31 patients with various clinical stages and histologic subtypes [110]. They uncovered distinct CAF subtypes, and *INHBA–FAP*-high cell populations were predictors of poor clinical prognosis.

### Liver cancer

#### Tumorigenesis

Despite the strong association between cirrhosis and HCC and its high medical relevance, the causal relationship between fibrosis and HCC development remains poorly understood and therapeutically underexplored [172]. Nearly all in vivo evidence and findings on the role of HSCs remain controversial. Filliol et al. [173] performed scRNA-seq of hepatic stellate cells (HSCs) from fibrotic mouse liver and snRNA-seq of HSCs from normal cirrhotic human livers to reveal the functions of HSCs during hepatocarcinogenesis. Signatures based on the differentially expressed genes (DEGs) were able to reliably identify more quiescent and activated mouse and human HSC subpopulations. Quiescent and cytokine-producing HSCs enriched for hepatocyte growth factor protected against hepatocyte death and HCC development. In contrast, activated myofibroblastic HSCs enriched for type I collagen, promoted proliferation and tumor development. An increased HSC imbalance between cytokine-producing HSCs and myofibroblastic HSCs during liver disease progression was associated with increased HCC risk in patients [173].

#### Treatment

The relationships between the immune phenotypic characteristics of innate lymphoid cells (ILCs) and HCC remain unclear. He et al. performed scRNA-seq on sorted hepatic ILCs from human patients with HCC and found that targeting inducible T-cell costimulator (ICOS) and its downstream effector HSP70 in ILC2s suppressed tumor growth and remodeled the immunosuppressive tumor microenvironment [174]. Liu et al. performed scRNA-seq on HCC tumors and adjacent normal tissues obtained from six ICB nonresponders. They discovered that the hypoxic microenvironment promoted SPP1 expression, and *SPP1*^+^ macrophages interacted with CAFs to stimulate extracellular matrix remodeling and promoted tumor immune barrier (TIB) structure formation, thereby limiting immune infiltration into the tumor core. Preclinically, blockade of SPP1 or macrophage-specific deletion of *Spp1* in mice led to enhanced efficacy of anti-PD-1 treatment in mouse liver cancer, accompanied by reduced CAF infiltration and increased cytotoxic T-cell infiltration [102]. Yuen et al. analyzed tumor-infiltrating T cells by flow cytometry and scRNA-seq. Based on the CD8^+^ T-cell infiltration level, they characterized tumors with different genotypes into cold and hot tumors. The single anti-PD-1 treatment appeared to be effective in HCCs with genetic mutations driving hot tumors, while combined anti-PD-1 and sorafenib treatment may be more appropriate for HCCs with genetic mutations driving cold tumors [175].

#### Prognosis

Song et al. performed scRNA-seq on 41,698 immune cells from seven pairs of HBV/HCV-related HCC tumor and nontumor liver tissues and identified one subset of CD8^+^ T cells with the high secretion of XCL1 that correlated with better prognosis [176]. He and colleagues used *k*-means clustering based on normalized abundances and identified seven distinct TME subtypes of HCC (TME1–7). Tumors of the TME2 and TME5 subtypes exhibited a macrophage-dominated and lymphocyte-depleted microenvironment and conferred the worst prognosis. In contrast, the TME7 subtype conferred the most favorable prognosis on their constituent tumors and exhibited high proportions of cytotoxic T lymphocytes, central memory T cells, and CD20^+^ B cells and low macrophage content [177]. Zhou et al. carried out scRNA-seq to analyze the transcriptomic profile of traced Prom1^+^ cells. By revealing the genetic profile of the Prom1 lineage, they found that the signature-high group had a significantly worse prognosis than the signature-low group in patients with HCC [178]. Another study revealed that high levels of COL1A1, ITGA2 and YAP were associated with poor prognosis in liver cancer patients [179].

### Breast cancer

#### Tumorigenesis, progression, and metastasis

The cell of origin (COO) in *BRCA1* mutant breast cancer is not clear, and the process of *BRCA1* mutant breast cancer development has not been fully elucidated. Based on RNA-seq and WES, Hu et al. identified that the impaired differentiation process of normal luminal cells in *BRCA1* mutation carriers might contribute to tumorigenesis. Moreover, the expression of *TP53* and *BRCA1* was decreased in luminal progenitor cells from normal breast tissue in *BRCA1* mutation carriers, which might trigger the basal/mesenchymal transition of luminal progenitors and might result in basal-like tumor development [180]. As the mechanisms governing seeding in distal tissues are poorly understood, Davis et al. established a robust method for the identification of global transcriptomic changes in rare metastatic cells during seeding using scRNA-seq and PDX models of breast cancer. The authors identified mitochondrial OXPHOS as the top pathway upregulated in micrometastases and found that pharmacological inhibition of OXPHOS substantially attenuated lung metastasis, showing that OXPHOS was functionally critical for metastatic spread [74]. Sun et al. conducted bulk RNA sequencing and scRNA-seq on both mammary gland cells and mammary tumor cells isolated from *Brca1* knockout mice. Of the candidate markers for *BRCA1* mutant tumors, we discovered and validated one oncogene *Mrc2,* whose loss could reduce mammary tumor growth in vitro and in vivo [181]*.*

#### Treatment

It remains unclear how stromal p38 signaling shapes the metastatic TME and affects tumor immunity in metastatic breast cancer. Faget et al. utilized a stromal labeling approach and scRNA-seq to identify targets that further increased the efficacy of p38MAPKα inhibitors (p38is). The combination of a p38i, anti-OX40, and cytotoxic T-cell engagement cured mice with metastatic disease and produced long-term immunologic memory [182].

#### Drug resistance

Triple-negative breast cancer (TNBC) is an aggressive subtype that frequently develops resistance to chemotherapy. Regardless of whether the resistance is caused by the selection of rare preexisting clones or through the acquisition of new genomic aberrations, Kim et al. [183] applied single-cell DNA and RNA sequencing in addition to bulk exome sequencing to profile longitudinal samples from 20 TNBC patients during NAC. The results indicated that resistant genotypes were preexisting and adaptively selected by NAC, while transcriptional profiles were acquired by reprogramming in response to chemotherapy in TNBC patients. The preexistence of chemoresistant genotypes in the tumor mass indicates that there may be diagnostic opportunities for detecting chemoresistant clones in TNBC patients prior to the administration of NAC to predict which patients are likely to benefit from chemotherapy and even raise the possibility of therapeutic strategies to overcome chemoresistance.

### Esophageal cancer

#### Treatment

Esophageal cancer is one of the most lethal cancers worldwide for human health because of its high morbidity and poor prognosis [184, 185]. Immunotherapy, the strategy to enhance the efficacy and specificity of the immune cells to suppress cancer progression, is a hot research area in cancer therapy including esophageal cancer [185]. Chen et al. [186] performed scRNA-seq analysis on five tumor samples and five corresponding nonmalignant samples from ESCC patients. The results revealed the potential role of LAG3 and HAVCR2 as checkpoint molecules for immunotherapy in ESCC.

#### Prognosis

Zhang et al. [187] investigated the composition of ESCC tumors based on 208,659 single-cell transcriptomes derived from 60 individuals. They found that high expression levels of the mucosal immunity-like (Mucosal) program in the tumor were strongly associated with the amount of effective infiltrating immune cells such as T follicular helper type 1 (T_FH_1), GC-B cells, and cDCs, and the researchers found that patients with high expression of the Mucosal program may have higher antitumor immunity and thus a better prognosis. The authors further identified that *CXCL17*, *AGR2,* and *MUC20* within the program were the markers that were best associated with ESCC survival.

### Ovarian cancer

#### Treatment

Malignant abdominal fluid (ascites) frequently develops in women with advanced HGSOC and is associated with drug resistance and a poor prognosis. Izar et al. used scRNA-seq to profile ~ 11,000 cells from 22 ascites specimens from 11 patients with HGSOC and found the JAK/STAT pathway was activated in both malignant cells and CAFs. The JAK/STAT inhibitor JSI-124 had potent antitumor activity in primary short-term cultures and PDX models of HGSOC [188]. Another study further suggested that EMT or JAK/STAT inhibitor combination therapy might enhance the treatment of HGSOCs [189]. Xu et al. [108] found that the immune coinhibitory receptor *TIGIT* was highly expressed on exhausted cytotoxic CD8^+^ T cells (CD8^+^ T_EX_) and that TIGIT blockade could significantly reduce ovarian cancer tumor growth in mouse models.

#### Prognosis

Previous bulk gene expression analysis on HGSTOC identified 4 molecular subtypes: the mesenchymal, immunoreactive, differentiated, and proliferative HGSTOCs. Stratification of patients according to these molecular subtypes failed to demonstrate differences in response rates to various therapies in clinical trials [190]. Schwede1 et al. [191] found cell admixture affects the interpretation and reproduction of ovarian cancer molecular subtypes and gene signatures derived from bulk tissue. As various factors in the stroma profoundly affect the prognostic impact of molecular subtypes, elucidating the role of stroma in the TME and prognosis is important and necessary by single-cell analysis or microdissection of tumor samples. Olbrecht et al. [192] performed scRNA-seq of 18,403 cells unbiasedly collected from 7 treatment-naive HGSTOC tumors and identified 6 prognostic subclusters. Of them, mesothelial cells (FB_CALB2), myofibroblasts (FB_MYH11), transforming growth factor ß-driven cancer-associated fibroblasts (FB_COMP), tumor subcluster Tum_BAMBI and lymphatic endothelial cells (EC_PROX1), predicted poor outcome, while plasma cells (BC_IGHG1_PRDM1^high^) were associated with improved OS. Sumitani et al. [193] performed scRNA-seq of serous ovarian cancer cells from four different patients to determine the association of each tumor population with poor prognosis. Two of the four identified tumor cell populations (a cancer-initiating cell population and a population expressing CA125) survived the initial treatment and suppressed antitumor immunity and were associated with poor prognosis. High levels of EMT and cell cycle signatures were significantly related to poor OS in ovarian cancer [189]. Xu et al. [108] further found that tumor cells were characterized by a set of EMT-associated gene signatures, from which the combination of *NOTCH1*, *SNAI2*, *TGFBR1*, and *WNT11* was further selected as a gene panel to predict the outcomes of patients with HGSOC.

### Melanoma

#### *Cancermetastasis*

In multiscale analyses using scRNA-seq data from six different primary uveal melanomas, Pandiani et al. assessed ITH at the genome and transcriptome levels. Their findings identified that HES6 increases the aggressive potential and motile capacity of primary uveal melanoma both in vitro and in vivo. Further experiments indicated that HES6 might be a valid target to limit uveal melanoma cell proliferation and migration [81].

#### Disease monitoring

Relatlimab plus nivolumab (anti-LAG3 + anti-PD-1) has been approved by the FDA as a first-line therapy for stage III/IV melanoma, but its specific effects on the immune system are unknown. Huuhtanen et al. [194] evaluated blood samples from 40 immunotherapy-naive or prior immunotherapy-refractory patients with metastatic melanoma treated with anti-LAG3 + anti-PD-1 using single-cell RNA and T-cell receptor sequencing (scRNA + TCRαβ-seq) combined with other multiomics profiling. They revealed that adaptive NK cells and CD8^+^ T cells have the highest *LAG3* expression and were more numerous in responders. Anti-LAG3 + anti-PD-1 treatment expanded LAG3^+^ NK cells, CD8^+^ T cells, and CD4^+^ T cells in responding patients. Tregs expand in the periphery following anti-LAG3 + anti-PD1 therapy but become metabolically silent during treatment.

#### Treatment

Many patients derive no clinical benefit from ICIs, and the molecular underpinnings of such resistance remain elusive [142]. However, a previous scRNA-seq analysis provided a glimpse into primary and metastatic uveal melanomas ecosystems, and disclosed a regulatory T-cell phenotype, highlighting LAG3 as a potential candidate for immune checkpoint blockade [94]. Recently, Li et al. performed scRNA-seq on nine clinical specimens (five primary tumor and four metastasis samples) of a rare subtype of melanoma named acral melanoma. Immune cells associated with acral melanoma exhibit the expression of multiple checkpoints including PD-1, LAG-3, CTLA-4, V-domain immunoglobin suppressor of T-cell activation (VISTA), TIGIT, and the adenosine A2A receptor (ADORA2). VISTA was expressed in 58.3% of myeloid cells and TIGIT was expressed in 22.3% of T/NK cells. These findings provide targets for future clinical immunotherapies for acral melanoma [195]. These scRNA-seq studies demonstrate the promising therapeutic role of ICIs in melanoma.

## Clinical applications of scRNA-seq in leukemia

ScRNA-seq assists us evaluate how combinatorial patterns of gene mutations change transcriptomic signatures and cellular behaviors, and provides a unique opportunity to identify novel tumor-specific targets in leukemia [196]. It is mainly used in AML and acute lymphoblastic leukemia (ALL) (Table 5).

**Table 5:** Clinical significance of scRNA-seq in various liquid tumors

Tumor	Year	Species	Protocol	Accession number (custom database if available)	Clinical significance	References
Acute myeloid leukemia	2019	Human	Seq-Well	GSE116256	Primitive AML cells exhibited dysregulated transcriptional programs with co-expression of stemness and myeloid priming genes and had prognostic significance	[261]
	2021	Human	Smart-seq2	GSE126068	Targeting both BCL2 and CXCR4 signaling might be a therapeutic strategy	[197]
	2022	Human	10 × Genomics	phs000159	Erythroid-related pathways were inhibited by decitabine, and this was reversed at relapse	[198]
	2023	Human	10 × Genomics	Correspondence with authors	Revealed premature accumulation of chemoresistant AML cells during early hematopoiesis. The hematopoietic stem cell-like cells from the non-CR group expressed more LSC markers (CD9, CD82, IL3RA, and IL1RAP) than those from the CR group. Chemoresistant progenitor cells had impaired myeloid differentiation owing to the early arrest of hematopoiesis	[199]
	2023	Human	10 × Genomics	HRA001240	QSCs were involved in the chemoresistance and poor outcomes of AML. The CD52-SIGLEC10 interaction between QSCs and monocytes might contribute to immune evasion and poor outcomes. LGALS1 was identified as a promising target for chemoresistant AML, and an LGALS1 inhibitor could help eliminate QSCs	[200]
	2023	Human	10 × Genomics	GSE196045	NFIC was identified as a transcription factor that was important for myeloid differentiation as well as AML cell survival and as a potential therapeutic target in AML	[152]
	2023	Human	10 × Genomics	GSE185993	Chemotherapy induced a generalized inflammatory and senescence-associated response. Some progenitor AML cells proliferated and differentiated with an expression of OXPHOS expression signature, while others were OXPHOS (low) miR-126 (high) and displayed high stemness and quiescence features	[127]
	2023	Human	10 × Genomics	Correspondence with authors	Identified a distinct LSC-like cluster with possible biomarkers in NK-AML (M4/M5). Provided an atlas of NK-AML (M4/M5) cell heterogeneity, composition, and biomarkers with implications for precision medicine and targeted therapies	[262]
	2023	Human	10 × Genomics	GSE213584	Identified unique C1Q^+^ macrophage-like leukemia cells. C1Q was identified as a marker for AML with adverse prognosis, orchestrated cancer infiltration pathway activity by communication with fibroblasts, and represents a compelling therapeutic target for EMI	[263]
Acute lymphoblastic leukemia	2020	Human	10 × Genomics	GSE134759	Monocyte abundance was predictive of pediatric and adult B-ALL patient survival. Human B-ALL cells promoted the emergence of CD16^+^ nonclassical monocytes ex vivo. Anti-CSF1R therapy enhanced the targeted treatment of Ph^+^ B-ALL models in vivo	[204]
	2021	Human	Smart-seq2	GSE161901	Combination therapies targeting diverse oncogenic states and the immune ecosystem seem most promising to successfully eliminate tumor cells that escape treatment through coexisting transcriptional programs	[203]
	2021	Human	10 × Genomics	EGAS00001004027	Multiple mechanisms leading to acquired *CD19* mutations contributed to CD19 loss and relapse on blinatumomab treatment. *CD19* ex2part alternative splicing levels were found to be a new biomarker predictive of blinatumomab resistance or failure	[202]
	2022	Human	10 × Genomics	HRA000489	The leukemic cells at relapse tended to take on poorly differentiated states, whereas the changes in the residual cells were more complicated. Inhibition of the hypoxia pathway sensitized leukemic cells to chemotherapy	[201]

### AML

#### Treatment and therapeutic monitoring

The cause of relapse is thought to be the persistence of leukemia-initiating cells (LICs) following treatment in AML. Stetson et al. [197] assessed RNA-based changes in LICs from matched samples taken at diagnosis and relapse using scRNA-seq. They demonstrated that targeting both BCL2 and CXCR4 signaling might help overcome therapeutic challenges related to AML heterogeneity.

The molecular mechanisms underlying decitabine response remain incompletely understood in myelodysplastic syndrome (MDS) and AML. Upadhyaya et al. [198] performed scRNA-seq on total bone marrow aspirate cells from 10 patients collected on days 0 and 10 of decitabine treatment. They found that decitabine induced global, reversible hypomethylation after 10 days of therapy in all patients, which was associated with induction of interferon-inducible pathways, expression of endogenous retroviral elements, and inhibition of erythroid-related transcript expression. Erythroid-related pathways were inhibited by therapy, which was reversed at relapse.

#### Drug resis0tance

Chemoresistance and relapse are the leading causes of AML-related deaths. Cheng et al. [199] used scRNA-seq to analyze the genetic profiles of 28,950 AML cells from 13 AML patients and found that chemoresistant AML cells prematurely accumulated during early hematopoiesis. Hematopoietic stem cell‐like cells from the non‐complete response (CR) group expressed more LSC markers (*CD9*, *CD82*, *IL3RA*, and *IL1RAP*) than those from the CR group. Chemoresistant progenitor cells had impaired myeloid differentiation owing to the early arrest of hematopoiesis.

A study revealed uncovered that miR-126^high^ LSCs were enriched at diagnosis and at relapse in chemotherapy-refractory AML and displayed enforced stemness and quiescence features, and these cells promoted chemotherapy resistance [127]. Another study produced similar results by dissecting the cellular states in bone marrow samples from primary refractory AML patients or those who relapsed soon after therapy through scRNA-seq. A subpopulation of quiescent stem-like cells (QSCs) was found to be involved in the chemoresistance and poor outcomes of AML [200].

### ALL

#### Treatment and therapeutic monitoring

The understanding of the resistance elicited in minimal residual disease (MRD) is limited due to the rarity and heterogeneity of the residual cells. Zhang et al. [201] assessed 161,986 single-cell transcriptomes to analyze the dynamic changes in B-cell acute lymphoblastic leukemia (B-ALL) at diagnosis, the development of residual, and relapse. In contrast to those at diagnosis, the leukemic cells at relapse tended to shift to poorly differentiated states, whereas the changes that occurred in the residual cells were more complicated. Both in vitro and in vivo models demonstrated that inhibition of the hypoxia pathway sensitized leukemic cells to chemotherapy. Another study revealed that the *CD19* ex2part splice variant is a new biomarker for predicting blinatumomab therapy failure [202].

#### Drug resistance

The resistance mechanisms in relapsed/refractory early T-cell progenitor acute lymphoblastic leukemia (ETP-ALL) carrying activating NOTCH1 mutations are unclear. Anand et al. [203] performed scRNA-seq on malignant and microenvironmental cells and identified 2 highly distinct stem cell-like states that were critically different in terms of cell cycle and oncogenic signaling. The fast-cycling stem cell-like leukemia cells demonstrated Notch activation and were effectively eliminated in patients by Notch inhibition, whereas the slow-cycling stem-cell like cells were Notch-independent and relied on PI3K signaling. These cells promoted an immunosuppressive leukemia ecosystem, accompanied by the clonal accumulation of dysfunctional CD8^+^ T cells.

#### Prognosis

Based on scRNA-seq and protein-based data of human B-ALL bone marrow and peripheral blood, Witkowski et al. found that CD16^+^ nonclassical monocytes may represent the majority of circulating and bone marrow monocytes, and they were found to be associated with inferior treatment outcomes [204]. Another study found a relapse-enriched B-cell subset was associated with poor prognosis, implicating the transcriptomic evolution during disease progression [205].

## Clinical utilities of scRNA-seq in lymphoma

Lymphoma has its own characteristics that differ from those of solid tumors and leukemia. In addition to the same clinical applications as other tumors, scRNA-seq can also aid in subtyping lymphoma (Table 6).

**Table 6:** Clinical significance of scRNA-seq in various lymphoma

Malignancies	Year	Species	Protocol	Accession number (custom database if available)	Clinical significance	References
Diffuse large B cell lymphoma	2019	Mouse	Smart-seq2	Correspondence with authors	Identified a “superphagocytic” macrophage subset that expressed CD36/FCGR4, and defined a novel mechanism through which high-dose alkylating agents promote macrophage-dependent lymphoma clearance	[228]
	2020	Human	10 × Genomics	GEO: GSE139891	Identified multiple functionally linked subpopulations. Effectively provided a single cell-COO for ∼80% of DLBCLs and identified novel prognostic subgroups of DLBCL	[213]
	2022	Mouse	GEXSCOPE® platform	Correspondence with authors	Combination treatment with dacinostat (LAQ824) and a c-Fos inhibitor more potently inhibited tumor cells both in vitro and in vivo	[264]
	2022	Human	10 × Genomics	CNGBdb: CNP0001940	TME cells may promote DLBCL activation/survival through CD40- and BAFF-mediated signals. Coinhibitory signals through TIM3 and TIGIT may drive T-cell exhaustion in DLBCL. HBV infection likely contributes to malignant cell survival/immune evasion in DLBCL	[216]
	2022	Human	10 × Genomics	GEO: GSE182436	Expression of both PD-1 and A2aR potentially defined a subset of much more dysfunctional CD8+ T cells and was associated with inferior outcomes	[265]
Follicular lymphoma	2022	Human	10 × Genomics	EGA: EGAD00001008311	Largely updated the NHC taxonomy in human LNs, performed an analysis of disease status, and provided a rich resource and deeper insights into LN and lymphoma biology to advance lymphoma management and therapy	[112]
Mantle cell lymphoma	2020	Human	10 × Genomics	Correspondence with authors	The drug resistance mechanisms of different cell clusters included drug metabolism, DNA damage repair, apoptosis, and survival promotion	[226]
	2021	Human	10 × Genomics	EGA: EGAS00001005019	Due to the 17q gain, BIRC5/survivin expression was upregulated in resistant MCL tumor cells, and targeting BIRC5 resulted in marked tumor inhibition in preclinical models	[227]
B-cell lymphoma	2022	Human	10 × Genomics	EGA: EGAS00001005356	*TIGIT* blockade alone improved the antitumor function of CAR-T cells	[75]
Burkitt lymphoma	2022	Mouse	10 × Genomics	GEO: GSE190598	Paclitaxel promoted the clearance of lymphoma by directly evoking the phagocytic capability of macrophages. Activation of Src family tyrosine kinase signaling in macrophages by paclitaxel promoted phagocytosis against NHL cells	[225]
Peripheral T cell lymphoma	2022	Mouse	10 × Genomics	GEO: GSE166673	*Vav1-Myo1f* induces the development of GATA3^+^ Th2-like PTCL and accumulation of TAMs. TAMs can be targeted for the treatment of high-risk PTCL	[266]
	2022	Cell line	10 × Genomics	GEO: GSE204876	PTCL had distinct *DNMT3A* mutation patterns and prognostic outcomes. *DNMT3A* mutations were associated with an activated, cytotoxic phenotype in the PTCL-TBX21 subtype	[230]
Angioimmunoblastic T cell lymphoma	2022	Human and mouse	10 × Genomics	ENA: ERP138895 or EGA: EGAS00001006401	Tet2-deficient immune cells functioned as a niche for AITL development. ACH-derived GCB cells potentially undergo independent clonal evolution and support tumorigenesis in AITL via the CD40-CD40LG axis	[134]
Adult T cell leukemia-lymphoma	2021	Human	10 × Genomics	NBDC: JGAS000301	Identified possible molecular mechanisms of multistep tumorigenesis and revealed the transcriptomic changes in identical infected clones over time during multistep tumorigenesis	[267]
	2022	Human	10 × Genomics	GEO: GSE195674	Identified a novel subset of CAFs, suggesting a potential target for targeted therapy to enhance treatment	[268]
Cutaneous T cell lymphoma	2019	Human	10 × Genomics	Correspondence with authors	*FOXP3* was identified as the most important factor to predict early disease in patients with CTCL. Transcriptome differences within a clonal tumor can be used to predict disease stage and thereby offer guidance for therapy	[73]
	2021	Human	10 × Genomics	GSE173205	Identified a specific panel of biomarkers that might be used for monitoring MF disease progression	[217]
	2022	Human	10 × Genomics	GSE124899 and GSE14658	New cellular clusters after progression of the therapy notably exhibited increased expression of the transcriptional factor *FOXP3*, raising the potential implication of an evolving mechanism of immune evasion	[209]
	2022	Human	10 × Genomics	GSE182861	Provided unprecedented new insights into MF/SS pathogenesis by reporting the transcriptional profile of malignant T-cell clones in the skin and blood of individual patients	[269]
	2022	Human	10 × Genomics	GSE197619	Identified putative precancerous circulating clonal CD4^+^ T-cell populations in patients with CTCL. The therapeutic potential of targeting CD82 and JAK was reported	[130]
	2022	Human	10 × Genomics	NCBI BioProject database PRJNA754592	Provided the first comprehensive compendium of genomic alterations in tCTCL, and identified potential prognostic signatures and novel therapeutic targets for a case of incurable T-cell lymphoma	[131]
	2022	Human	10 × Genomics	dbGaP: phs002933.v1.p1	Supported further study of *KIR3DL2* expression and CD8 immune populations as predictive biomarkers of pembrolizumab response in patients with Sézary syndrome	[224]
T-cell lymphoma	2022	Zebrafish	10 × Genomics	GSE166646	Described the oncogenicity of *IRF4 *in vivo, its potential effects on T-cell development, and clonal evolution using a zebrafish model. IRF4-driven tumors were sensitive to a BRD inhibitor	[270]
Classic Hodgkin lymphoma	2021	Human	10 × Genomics	EGA: EGAS00001005541	This study strongly suggested the pathogenic importance of the CXCL13/CXCR5 axis and that PD-1^+^CXCL13^+^ T cells are a treatment target in LR-CHL	[271]

### Diagnosis

Cancers are traditionally diagnosed by their tissue of origin and histologic features [206]. ScRNA-seq, as the earliest and best-established single-cell sequencing technique, has been used to identify diagnostic biomarkers [206]. Gaydosik et al. [77] identified a 17-gene expression signature (*ANP32*, *BPPIA*, *ATP5C1*, *PSMB2*, *DUT*, *RAN*, *HMGN1*, *RANBP1*, *HN1*, *SET*, *NPM1*, *SMC4*, *NUSAP1*, *STMN1*, and *PCNA*) common to all five tumors tested. The authors validated the protein coexpression of three of the genes (*PCNA*, *ATP5C1*, and *NUSPA1*) with *TOX* in multiple patients with advanced-stage CTCL. Thus, these genes have the potential to be diagnostic markers for CTCL. Jonak et al. [207] performed scRNA-seq on two 6-mm skin punch biopsies from a 33-year-old patient with concurrent mycosis fungoides (MF) and primary cutaneous follicle center lymphoma (PCFCL) and revealed a type-2 immune skewing in MF, while PCFCL lesions generally exhibited a more type-1 immune phenotype, consistent with its indolent behavior. This result indicated the existence of two clonal malignancies in the skin of a single patient that occurred at the same time, but developed in distinct skin lesions in a nonoverlapping manner; these features were most consistent with the diagnosis of discordant lymphoma, and the case provides proof of concept that scRNA-seq can be applied to diagnose primary cutaneous lymphomas. Li et al. [208] identified four genes (*CYTOR*, *CXCL13*, *VCAM1*, and *TIMD4*) that were explicitly expressed in malignant T cells that may be novel markers for subcutaneous panniculitis-like T-cell lymphoma (SPTCL). Ren et al. [130] performed integrative paired scRNA-seq and single-cell TCRαβ sequencing (scTCRseq) analyses of CD4^+^ T cells from 11 MF/SS patients. They found that 7 genes (i.e., *AC133644.2*, *UBXN11*, and *ADTRP*) were upregulated while 28 genes (i.e., *TRBC1*, *AIRE*, and *TPO*) were downregulated in SS relative to MF, suggesting that these genes could be biomarkers for diagnosis and predicting prognosis. However, another study identified that the *AIRE* gene was expressed in 58% of malignant cells versus 8.7% of nonmalignant cells across samples and was the most highly upregulated gene in SS [209].

### Subtyping

The COO classification has identified two subtypes of diffuse large B-cell lymphoma (DLBCL): GCB and activated B-cell-like (ABC) DLBCL, with GCB cases characterized by a better prognosis than ABC cases [210–212]. As the COO classification represents an oversimplification of the complex dynamics of the proliferation, trafficking, and differentiation of B cells within the germinal center, Holmes et al. [213] applied genome-wide single-cell (sc) RNA profiling to further dissect the heterogeneity of germinal center B cells. The authors explored the dynamics of germinal center B-cell development beyond the known DZ and LZ compartments and identified five stages: DZ cells (*CXCR4*, *AICDA*), intermediate cells (*CXCR4* and *CD83*), LZ cells (*CD83* and *BCL2A1*), plasmablasts (PBL; *PRDM1*and *IRF4*), or precursor memory B (PreM; *CCR6*) cells. Based on the five stages, 13 sc-COO subtypes (DZ a, DZ b, DZ c, INT a, INT b, INT c, INT d, INT e, LZ a, LZ b, PreM, PBL a and PBL b) were identified, and they provided sc-COO for ∼80% of DLBCLs. In addition to DLBCL typing based on B cells, a novel DLBCL typing using scRNA-seq has also emerged beyond COO and genotypic classes. For a further step, survival analysis indicated Groups II and IV had the worst and best survival outcomes, respectively while the remaining three groups had an intermediate prognosis in a merged dataset containing National Cancer Institute [NCI]-DLBCL data and (British Columbia Cancer Agency [BCCA]-DLBCL data. The results indicated that the sc-COO classification can identify clinically relevant subgroups within GCB- and ABC-DLBCLs, as well as DHITsig-positive cases. In another study, the authors identified 44 distinct cellular states from all 12 cell types, ranging from two to five states per cell type. Then, they applied EcoTyper to reveal nine multicellular ecosystems in DLBCL based on the 44 cellular states, namely, lymphoma ecotypes (LEs). Although the results of this study reclassified DLBCL based on cell states and ecosystems, future studies will be needed to further characterize the spatial topology and interactions within LEs and the molecular switches that mediate therapeutic responsiveness and resistance in DLBCL [214].

In addition to DLBCL, Liu et al. also established a binary subtyping scheme for CTCL based on the molecular features of malignant T cells and their protumorigenic microenvironments [78]. The cytotoxic effector memory T-cell (T_CyEM_) group, which displayed a cytotoxic effector memory T-cell phenotype, showed more M2 macrophage infiltration, while the T_CM_ group, featuring a central memory T-cell phenotype and worse patient outcomes, was infiltrated by highly exhausted CD8^+^ reactive T cells, B cells and Tregs with suppressive activities.

### Disease monitoring

Noninvasive monitoring of disease status, prognosis and treatment response, and early detection of relapse are preferred in clinical practice, as biopsy is unfortunately impractical at times [215]. Ye et al. [216] compared the malignant cell compartment in one pair of samples collected at diagnosis and relapse. The scRNA-seq data suggested that selective outgrowth of cells with an activated MAPK signaling program might be associated with relapse in the DLBCL patient. Borcherding et al. [209] extensively mapped the transcriptomic variations in approximately 50 000 T cells of both malignant and nonmalignant origins using single-cell mRNA and TCR sequencing of peripheral blood immune cells in patients with SS. New cellular clusters identified after progression on therapy notably exhibited increased expression of the transcription factor *FOXP3,* a master regulator of Treg function, suggesting the potential of an evolving mechanism of immune evasion. Rindler and his colleagues [217] revealed a specific panel of biomarkers that might be used for monitoring MF disease progression. Despite considerable interindividual variability, lesion progression was uniformly associated with the downregulation of the tissue residency markers *CXCR4* and *CD69,* the heat shock protein *HSPA1A,* the tumor suppressors and immunoregulatory mediators *ZFP36* and *TXNIP,* and interleukin 7 receptor (*IL7R*) within the malignant clone but not in benign T cells.

### Treatment

Traditional treatments for lymphoma include chemotherapy with or without radiotherapy, surgery, and bone marrow transplantation [218, 219]. In recent years, targeted therapy and immunotherapy have gradually emerged as options and have been applied in clinical trials, with encouraging achievements against malignant lymphoma [218, 220–222].

Some studies have explored the application of scRNA-seq to develop targeted therapy. Targeted therapies can be divided into two categories. Some targeted therapies target specific molecules, and others target distinct cell populations or subpopulations. Fujisawa et al. [134] performed scRNA-seq of 5 human AITL tumors and 3 homeostatic lymph node (HLN) samples, and an in silico network analysis using the scRNA-seq data identified CD40-CD40LG as a possible mediator of GCB and tumor cell cluster interactions. Therefore, blockade of the CD40-CD40LG axis by administering an anti-Cd40lg antibody suppressed tumor growth. Ren et al. [130] found that CD82 regulates CTCL proliferation and apoptosis through the JAK/STAT and AKT/PI3K pathways and revealed the therapeutic potential of targeting CD82 and JAK, which endow malignant CTCL cells with survival and proliferation advantages. A DLBCL study identified the CD74-MIF interaction as the most significant interaction between B cells and the other three types of immune cells (T cells, macrophages, and DCs) [85]. This same ligand‒receptor interaction also resulted in significant upregulation of MIF in malignant T cells and interactions of malignant T cells expressing MIF with macrophages and B cells expressing CD74 in CTCL [131]. These findings suggest the utility of targeting the CD74-MIF interaction with therapies for tCTCL.

Targeting macrophages in hematological malignancies is a promising approach since these cells either support or inhibit tumor growth depending on their phenotypes and functions [98, 223]. In one study, Cao et al. [75] used paclitaxel to substantially increase the anticancer efficacy of CD47-targeted therapy in late-stage non-Hodgkin lymphoma (NHL) by activating Src family tyrosine kinase signaling in macrophages. Paclitaxel re-enabled programmed cell removal (PrCR) by not only directly stimulating the phagocytic capacity of bone marrow macrophages but also reversing the phagocytosis-inhibitory TME through the suppression of TAM populations directly linked to NHL progression. In addition to TAMs, CD8^+^ T cells are another target population. Steen et al. [214] divided CD8^+^ T cells into five state (S1-S5) subpopulations. The transcriptomic and spatial characterization linked CD8^+^ T cells in the S1 subpopulation to a previously described CXCR5^+^ CD8^+^ T cell state. Patients harboring large numbers of CD8^+^ T cells in the S1 subpopulation experienced significantly longer survival in the RB-CHOP arm (bortezomib added to standard R-CHOP therapy) than those in the R-CHOP arm in terms of both OS and PFS.

Immunotherapies are divided into two categories based on the mechanism of action. Some are designed to block the immune evasion of tumor cells, and these therapies are represented by ICIs, most of which target PD-1, PD-L1, cytotoxic T lymphocyte-associated protein 4 (CTLA-4), and other related checkpoints. Other immunotherapies are designed to enhance the ability of immune cells to attack tumor cells, and these therapies are represented by cellular treatment with chimeric antigen receptor (CAR)-T cells [218]. Su et al. [224] found that responses to pembrolizumab were associated with lower expression of *KIR3DL2* within the Sézary cell population, suggesting that treatment with anti-KIR3DL2 drugs such as lacutamuab synergized with pembrolizumab therapy. Another study found that both PD-1/PD-L1 and CD73/A2aR signaling mediated the immunosuppressive microenvironment in DLBCL. A combination of treatments targeting the immunosuppressive PD-1/PD-L1 axis with CD73/A2aR inhibitors may provide additional clinical benefits and may overcome primary and secondary resistance to PD-1/PD-1L1 blockade.

Jackson et al. [225] performed a sequential analysis of manufactured and infused CAR-T cells using single-cell RNA and protein expression data for the first time to investigate the mechanisms linked to patient response to CAR-T-cell therapy. CAR-T cells exhibited significant heterogeneity across time points (product, Day 14, and Day 30), cell-cycle phases, cell types, and patients. The authors noted that the CAR-T cells evolved toward a nonproliferative, highly differentiated, and exhausted state, with an enriched exhaustion profile marked by high *TIGIT* expression observed in CAR-T cells from patients with a poor response; that is, there was a shift in the predominant profiles of CD8^+^ CAR-T cells from CD45RA^hi^CCR7^hi^CD127^hi^CD62L^hi^CD25^hi^ cells to CD45RO^hi^CD28^hi^CD69^hi^CD27^hi^PD-1^hi^ and CD45RA^hi^CD57^hi^CD69^hi^PD-1^hi^ cells upon infusion. CD8^+^ TIGIT^+^ CAR-T cells had greater dysfunctional scores than TIGIT^−^ cells, upregulated *TOX* expression and upregulation of many of the same exhaustion-related genes that were differentially expressed between response groups and had a higher surface expression of all exhaustion markers tested, including PD-1. These findings revealed for the first time that *TIGIT* inhibition alone could improve CAR-T-cell efficacy in mouse models, as well as in models treated with a clinically relevant monoclonal blocking antibody.

### Drug resistance

Drug resistance is one of the principal reasons for the failure of anti-infection drugs and cancer chemotherapy [226]. Tumor heterogeneity is the main driving force of drug resistance [136].

Wang et al. [226] performed scRNA-seq on bone marrow samples from a patient with relapsed mantle cell lymphoma (MCL). The results indicated that the major immune escape mechanisms of MCL included inhibition of perforin activity, decreased immunogenicity, and direct inhibition of apoptosis and cell killing mediated by type I (CCND1^+^CD79A^+^TNFRSF13C^+^) and II B (CCND1^+^CD79A^+^) cells. However, type I B cells displayed greater proliferation and differentiation potential than other clusters, thus indicating that these cells had greater potential for immune escape and the activation of drug resistance mechanisms and might be useful drug targets in the future.

The TME, which comprises cellular and noncellular components, plays a crucial role in drug resistance [7]. Zhang et al. [227] performed sequential scRNA-seq of 21 specimens collected at baseline, during treatment, and/or at disease remission/progression from three ibrutinib-responsive patients and 2 nonresponsive patients to further explore the molecular heterogeneity and the mechanism of drug resistance in refractory MCL. A cell-to-cell communication analysis revealed that complex interactions between MCL cells and the TME might largely influence therapeutic resistance, warranting the development of strategies to promote the anti-lymphoma activity of the TME. Lossos et al. [228] established PDXs of double-hit lymphoma (DHL) to more faithfully model human aggressive lymphomas. According to the study, rituximab resistance within the bone marrow was not present upon early engraftment but developed during lymphoma progression. Furthermore, this resistance required a high tumor cell:macrophage ratio and was overcome by multiple, high-dose alkylating agents. ScRNA-seq of the macrophages identified a “superphagocytic” subset that expressed CD36/FCGR4, suggesting that these cells were the primary effectors that mediated the activity of the single agent cyclophosphamide. These findings revealed a novel mechanism by which high-dose alkylating agents promoted macrophage-dependent lymphoma clearance.

### Prognosis

An accurate evaluation of the prognosis of cancer patients is important [129]. Currently, three types of prognostic factors identified by scRNA-seq have been reported in lymphoma. The first is gene markers. Abe et al. utilized multistep DEG analyses and revealed *LY6H* expression which was first described in mouse and human endothelial cells in FL. Increased expression of the markers *LY6H* and *LOX,* as well as *TDO2* and *REM1,* was associated with an unfavorable prognosis [112]. Zhao et al. [229] constructed prognostic models based on differentially expressed genes associated with CD8^+^ T_EX_ subpopulations, and six prognosis-related genes were obtained for model construction using multivariate Cox regression analysis (*GABRA3*, *HOXC8*, *RTN4R*, *CRLF1*, *BIRC3*, and *REXO5*). The prognostic model could identify high-risk DLBCL patients and aid clinical decision making. Borcherding et al. [73] showed the involvement of *FOXP3*^+^ malignant T cells in clonal evolution using scRNA-seq and the machine-learning reverse graph embedding approach: *FOXP3*^+^ T cells transitioned into *GATA3*^+^ or *IKZF2*^+^ (HELIOS) tumor cells in a patient with stage IVA SS. *FOXP3* was identified as the most important factor for the early prediction of disease in patients with CTCL; it and 19 other genes could predict the CTCL stage with approximately 80% accuracy.

The second type of prognostic factor is cell subpopulations. The sc-COO classification of DLBCL has been reported in the section of *subtyping* [213]. Another scRNA-seq analysis showed that *DNMT3A* mutations defined a cytotoxic subset associated with a significantly worse prognosis of PTCL-TBX21, this result can be used to further refine pathological heterogeneity in PTCL-NOS and suggests alternative treatment strategies for this subset of tumors [230].

The third prognostic factor is gene signatures, which usually consist of tens to hundreds of genes [231]. Liu et al. [78] conducted a hierarchical clustering analysis of 65 gene signatures and revealed four main meta-programs, which indicated that malignant T cells have similar behaviors across the heterogeneous transcriptional spectrum of CTCL tumors. Annotation of the top-ranking genes of the four meta-programs identified distinct functional signatures, including T-cell signaling and activation (meta-program 1: *HLA-DRB1*, *CD69,* and *MYC*; and meta-program 4: *ITK*, *FYN,* and *CBLB*), cell cycle (meta-program 2: *MCM7*, *PCNA,* and *BIRC5*) and cell metabolism (meta-program 3: *GAPDH*, *BUA52,* and *RPS3*). Notably, a high T-cell activation signature (meta-program 1 and meta-program 4) was associated with a favorable prognosis, while a high proliferation signature (meta-program 2) predicted a poor patient outcome.

## Conclusions

ScRNA-seq provides unprecedented mapping data for cancer analyses because it provides high-resolution transcript sequencing; it can be used for the discovery of new cell subsets and markers, the analysis of intratumor and intertumor heterogeneity, and studies of the tumor microenvironment, intercellular crosstalk, and lineage trajectories, and such studies have resulted in great progress in our understanding of cancer pathogenesis, diagnosis, prognosis, treatment, and drug resistance.

However, scRNA-seq has many disadvantages that limit its widespread application. First, although scRNA-seq has been the most mature and widely-used single-cell omics in cancer research, it can’t completely reflect cancer biology, such as the level of genomics, proteomics, epigenomics, etc. Second, scRNA-seq has high requirements for specimens, and can only use live cells as detection objects; specimens analyzed at different times will have associated batch effects. Third, enzymatically digesting cells to obtain single-cell suspensions is likely to kill cells and degrade RNA. This will also result in the loss of spatial and morphologic information. Fourth, not all nucleated cells are detected, the sequencing depth may be limited, and scRNA-seq data do not fully reflect the entire transcriptional landscape and genetic information of cancer samples. The heterogeneity of patient tumors and differences in detection platforms also increase the difficulty of result interpretation and decrease data uniformity. In addition, scRNA-seq is expensive and time-consuming, and data processing is complex.

Therefore, scRNA-seq technology still has much room for improvement in cancer research. In the future, researchers should focus on reducing the price, further improving single-cell isolation technology, improving throughput and sequencing depth, enhancing bioinformatics analysis pipelines, and expanding applications to utilize frozen and formalin-fixed paraffin-embedded (FFPE) tissues to improve the ease of use and accessibility. In addition to advances in scRNA-seq technology, scRNA-seq should also be combined with other omics technologies and artificial intelligence technology. ScRNA-seq-plus-genomics records the dynamics between gene mutations and expression; scRNA-seq-plus-proteomics reveals the relationship between transcript abundance and resulting protein content; scRNA-seq-plus-epigenomics helps understand the regulation of gene expression by chromatin structure or methylation; scRNA-seq-plus-metabolomics map and quantify the in sufficient detail to provide useful information about cellular function in highly heterogeneous cancers [66, 67]. Apart from the single-cell multi-omics, spatial multi-omics also should be paid attention to and be combined with scRNA-seq or other single-cell omics, which will help us deepen our understanding of the cell location and its various functions. Other cell-labeling technologies such as CRISPR/Cas9 [232] and nuclear hashing [57] have been combined with scRNA-seq. They can identify and characterize the effects of thousands of independent genetic perturbations in vivo on tissues or cells with different functions at single-cell resolution. Of course, the combination of more omics requires more samples, which is also a serious challenge in terms of money and ethics. The application of more technologies generates more data, which also raises the requirements for researchers or clinicians to analyze and apply the data, and puts higher demands on bioinformatics. As a result, scRNA-seq with various other omics and technologies needs to be more tightly integrated to reduce the cost and sample volumes. Moreover, artificial intelligence-based machine learning and data processing analyses should keep up with the scRNA-seq technology to provide researchers with more useful information that will improve the understanding of cancer. Lastly and most importantly, scRNA-seq should be combined with clinical needs to provide patients with better personalized and precise treatment options. As a relatively new technology that has not yet been widely used in clinical practice, in addition to the norms that need to be developed for the technology itself, the ethical norms for its use in clinical implementation also need to be formulated and standardized by experts.

## Data Availability

Attached data.

## Abbreviations

ABC: Activated B cell-like
ACH: Age-related clonal hematopoiesis
ADC: Adenocarcinoma
ADORA2: Adenosine A2A receptor
AITL: Angioimmunoblastic T cell lymphoma
ALL: Acute lymphoblastic leukemia
AML: Acute myeloid leukemia
ATLL: Adult T cell leukemia-lymphoma
B-ALL: B-cell acute lymphoblastic leukemia
BCCA: British Columbia Cancer Agency
BECs: Blood endothelial cells
BL: Burkitt lymphoma
CAFs: Cancer-associated fibroblasts
CAR: Chimeric antigen receptor
CAR-T: Chimeric antigen receptor T cell
cDC: Conventional dendritic cell
cDNA: Complementary DNA
CEL-seq: Cell expression by linear amplification and sequencing
CHL: Classic Hodgkin lymphoma
CNSL: Central nervous system lymphoma
COO: Cell of origin
CRC: Colorectal cancer
CSC: Cancer stem cell
CTCL: Cutaneous T cell lymphoma
CTCs: Circulating tumor cells
CTLA-4: Cytotoxic T lymphocyte-associated protein 4
CXCL13: Chemokine C-X-C motif ligand 13
CXCR5: C-X-C motif chemokine receptor 5
CyTOF: Cytometry by time of flight
DCs: Dendritic cells
DGC: Differentiated gastric cancer
DHL: Double-hit lymphoma
DLBCL: Diffuse large B cell lymphoma
DZ: Dark zone
eCAFs: Extracellular matrix CAFs
EMT: Epithelial-to-mesenchymal transition
ESCC: Esophageal squamous cell carcinoma
ETP-ALL: Early T-cell progenitor acute lymphoblastic leukemia
FACS: Fluorescence-activated cell sorting
FACT: Facilitates chromatin transcription
FDCs: Follicular dendritic cells
FFPE: Formalin-fixed paraffin-embedded.
FL: Follicular lymphoma
FS: FLASH-seq
FS-LA: FLASH-seq low-amplification
FS-UMI: FLASH-seq with UMIs
GAC: Gastric adenocarcinoma
GC: Germinal center
GCB: Germinal center B cell-like
GSRCC: Gastric signet-ring cell carcinoma
HB: Hepatoblastoma
HCC: Hepatocellular carcinoma
HDACi: Histone deacetylase inhibitor
HGSOC: High-grade serous ovarian cancer
HGSTOC: High-grade serous tubo-ovarian cancer
HLN: Homeostatic lymph node
HSCs: Hepatic stellate cells
HTS: High-throughput screening
iCAFs: Inflammatory CAFs
ICAM1: Intercellular adhesion molecule-1
ICB: Immune checkpoint blockade
ICIs: Immune checkpoint inhibitors
ICOS: Inducible T cell costimulator
IL7R: Interleukin 7 receptor
ILCs: Innate lymphoid cells
ITH: Intratumor heterogeneity
IVT: In vitro transcription
LCM: Taser-capture microdissection
LECs: Lymphatic endothelial cells
Les: Lymphoma ecotypes
LICs: Leukemia initiating cells
LKB1: Liver kinase B1
lncRNAs: Long non-coding RNAs
LR-CHL: Lymphocyte-rich classic Hodgkin lymphoma
LSCs: Leukemia stem cells
LTS: Long-term survivors
STS: Short-term survivors;LUAD: Lung adenocarcinoma
LZ: Light zone
MACS: Magnetic-activated cell sorting
MARS-seq: Massively parallel single-cell RNA-sequencing
mCAFs: Matrix cancer-associated fibroblasts
MCL: Mantle cell lymphoma
mCRC: Metastatic colorectal cancer
mDC: Myeloid dendritic cell
MDS: Myelodysplastic syndrome
MF: Mycosis fungoides
MFLNs: Metastasis-free lymph nodes
MHC: Major histocompatibility complex
MIA: Multimodal intersection analysis
miRNAs: MicroRNAs
MLN: Metastatic lymph nodes
MRCs: Marginal reticular cells
MRD: Minimal residual disease
MSI: Microsatellite instability
Mφ: Macrophages
NAC: Neoadjuvant chemotherapy
NCI: National Cancer Institute
NEC: Neuroendocrine carcinoma
NESCs: Nonendothelial stromal cells
NET: Neuroendocrine tumor
NFIC: Nuclear factor I-C
NGS: Next-generation sequencing
NHCs: Nonhematopoietic cells
NHL: Non-Hodgkin lymphoma
NPM1: Nucleophosmin 1
NSCLC: Non-small cell lung cancer
OS: Overall survival
p38is: P38MAPKα inhibitors
PBL: Plasmablast
PC: Peritoneal carcinomatosis
PCFCL: Primary cutaneous follicle center lymphoma
PCNSL: Primary central nervous system lymphoma
PD: Progressive disease
PD-1: Programmed cell death protein 1
pDC: Plasmacytoid dendritic cell
PDGC: Poorly differentiated gastric cancer
PD-L1: Programmed cell death-ligand 1
PDX: Patient-derived xenograft
PEL: Primary effusion lymphoma
PFS: Progression-free survival
PHATE: Potential of heat-diffusion for affinity-based trajectory embedding
piRNAs: Piwi-interacting RNAs
PrCR: Programmed cell removal
PreM: Precursor memory B
PTCL: Peripheral T cell lymphoma
QSCs: Quiescent stem-like cells
RD: Residual disease
RNA-seq: RNA sequencing
RT: Reverse transcription
sc: Single-cell
scATAC-seq: Single-cell Assay for Transposase-Accessible Chromatin using sequencing
SCC: Squamous cell carcinoma
scDNA-seq: Single-cell DNA sequencing
Sci-RNA-seq: Single-cell combinational indexing RNA sequencing
SCNAs: Somatic mutations as well as somatic copy number alterations
scRNA-Seq: Single-cell RNA sequencing
SCs: Stromal cells
scTCRseq: Single-cell TCRαβ sequencing
SPLiT-seq: Split-pool ligation-based transcriptome sequencing
SPTCL: Subcutaneous panniculitis-like T cell lymphoma
SRT: Spatially resolved transcriptomics
SS: Sézary syndrome
TACE: Transarterial chemoembolization
TAMs: Tumor-associated macrophages
TCR: T-cell receptor
tCTCL: Transformed CTCL
T_CyEM_: Cytotoxic effector memory T cell
TEX: Exhausted cytotoxic CD8 + T cells
TGS: Third-generation sequencing
Th1: T helper 1
TIB: Tumor immune barrier
TIME: Tumor immune microenvironment
TME: Tumor microenvironment
TNBC: Triple-negative breast cancer
Tregs: Regulatory T cells
TSO: Template-switching oligonucleotide
UM: Uveal melanoma
UMI: Unique molecular identifier
VISTA: V-domain immunoglobin suppressor of T cell activation
WES: Whole exome sequencing
WGS: Whole genome sequencing
YAP1: Yes-associated protein 1
yCRC: Young-onset colorectal cancer
